# Right handed chiral superstructures from achiral molecules: self-assembly with a twist

**DOI:** 10.1038/srep15652

**Published:** 2015-10-23

**Authors:** A Anuradha, Duong Duc La, Mohammad Al Kobaisi, Sheshanath V. Bhosale

**Affiliations:** 1 School of Applied Sciences, RMIT University, GPO Box 2476, Melbourne, Vic. 3001, Australia

## Abstract

The induction and development of chiral supramolecular structures from hierarchical self-assembly of achiral compounds is closely related to the evolution of life and the chiral amplification found in nature. Here we show that the combination of achiral tetraphenylethene (TPE) an AIE-active luminophore bearing four long alkyl chains *via* amide linkage allows the entire process of induction and control of supramolecular chirality into well-defined uniform right-handed twisted superstructures *via* solvent composition and polarity, i.e. solvophobic effect. We showed that the degree of twist and the pitch of the ribbons can be controlled to one-handed helical structure *via* solvophobic effects. The twisted superstructure assembly was visualised by scanning electron microscope (SEM) and transmission electron microscopy (TEM), furthermore, circular dichroism (CD) confirms used to determine controlled right-handed assembly. This controlled assembly of an AIE-active molecule can be of practical value; for example, as templates for helical crystallisation, catalysis and a chiral mechanochromic luminescent superstructure formation.

Iduction and control of supramolecular chiral assembly from achiral functional organic molecules presents a great challenge, to provide systems that can increase our understanding of some life processes, and afford potential applications in areas such as sensors, liquid crystals, and optical activity^1^,^2^,^3^,^4^. Supramolecular helical or twisted objects have been generally induced by the self-assembly of small organic molecules containing chiral groups such as bola-amphiphile, peptides, lipid bilayers, glucose and π-conjugated oligomers^5^,^6^,^7^,^8^,^9^. In some cases, twisted or helical fibres have been produced using homochiral molecules or a mixture of chiral and achiral molecules. This can also be achieved through the introduction of chiral molecular templates, where the molecular chirality is transferred to the handedness of the self-assembled helical structure^10^,^11^,^12^,^13^,^14^. Supramolecular chiral assembly by irradiation of azobenzene-containing achiral molecules with circularly polarized light have also been reported^15^,^16^,^17^. Only a few reports describe generation of helical nanostructures from achiral molecules such as amphiphilic cyanine dyes, cationic surfactants, asymmetric porphyrins and quinacridone and also through chiral control of interfacial tension among the few^18^,^19^,^20^,^21^,^22^,^23^,^24^. However, most of these small molecules suffer from aggregation induced quenching (ACQ) effects^25^, which is very important in the design of mechanochromic luminescent materials. The self-assembly and function of the helices from the photoluminescent achiral molecules are far from being investigated. Nevertheless, Zhao *et al*. describe the fabrication of twisted nanostructures by two achiral luminescent quinacridone (QA) derivatives from THF/ethanol mixes^24^.

The design and synthesis of achiral organic functional molecules which can assemble into an architectures with selective handedness and aggregation induced fluorescence (AIE) in the absence of chiral substances is an important step in understanding the role chirality plays within these systems^26^.

Recently, the unusual fluorescence properties of tetraphenylethylene (TPE) and its derivatives, has attracted great interest. These chromophores have weak or non-emissive nature as unassociated monomers, but they become strongly fluorescent upon aggregation i.e. AIE-effect^27^,^28^. AIE properties are of interest in diverse areas including fluorescence sensors^29^ and electroluminescent organic materials^30^. Recently, pH-dependent construction of nanofibres based on pyridyl-substituted TPE derivative and their luminescence properties were studied^31^. In another report, the self-assembly of TPE-based porphyrin derivatives that self-assembled into well-defined ring nanostructures was also reported, these results are similar to natural photosynthesis system-II^32^. Although TPE derivatives have been used to study the interaction of AIE molecules (AIEs) with DNA^33^, proteins^34^,^35^ or other biostructures^36^, the fabrication of supramolecular helical structures using achiral AIE-active TPE luminophore as building blocks has never been investigated.

## Result

**Figure 1:**
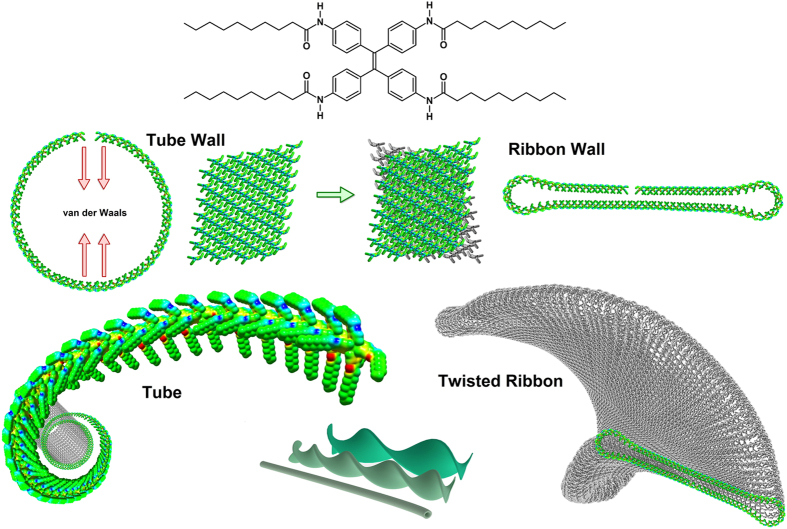
Hypothetic illustration of right-handed helical superstructures. The molecular chiral arrangement of achiral alkyl-TPE results in the formation of a cylindrical tubular microstructure with helical molecular arrangement, the walls of these cylindrical structure at a critical diameter collapse to improved morphological stability by maximizing hydrophobic interactions between the alkyl chains and assemble into right-handed twisted-ribbon, furthermore these ribbons can stack to form stable multilayered superstructures. The width of the ribbons and their stacking can vary as function of the dynamics and kinetics of the solvent evaporation/self-assembly process.

In this contribution, we report a chiral supramolecular nanotube and twisted ribbon morphologies based on hierarchically organized supramolecular microarchitectures (Fig. 1) from an achiral TPE derivative *via* solvophobic control. In this case, AIE active TPE, bearing alkyl-group *via* amide linkage (**alkyl-TPE**), possess three important features resulting in the formation of controlled twisted chiral assembly: (i) the π-π interactions between the aromatic TPE cores within a construct, (ii) long alkyl chains on the periphery of the TPE, which are designed to optimize the dispersive and van der Waals interactions, and (iii) hydrogen-bonding *via* amide functional groups. These interactions prevent crystallization and favour arrangement with directional growth of twisted superstructure as illustrated in Fig. 1. We believe this to be the first example of self-assembling systems where the entire process of induction and control of supramolecular chirality from achiral AIE-active luminophore proceeds *via* solvophobic control.

**Figure 2:**
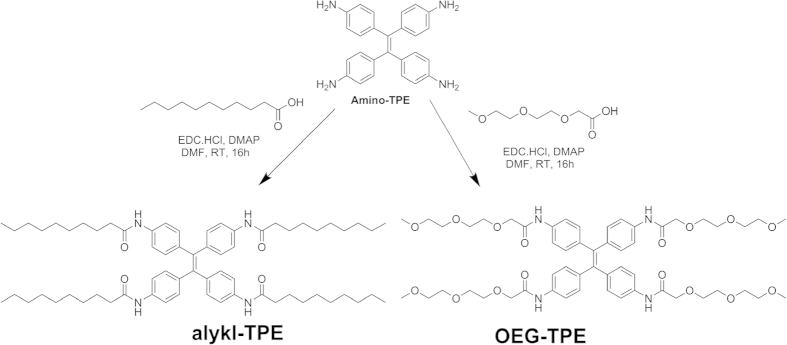
Synthesis of target derivatives.

Achiral compound **alkyl-TPE** was synthesized by an amide coupling reaction between an amine-TPE molecule^37^ and decanoic acid in the presence of 1-ethyl-3-(3-dimethylaminopropyl) carbodiimide (EDC) and 4-dimethylaminopyridine (DMAP). Following similar procedure **OEG-TPE** was synthesised from amine-TPE and OEG-acid and as shown in Fig. 2, for detail synthesis see Supplementary Information.

### UV-vis absorption and fluorescence spectroscopy

**Figure 3:**
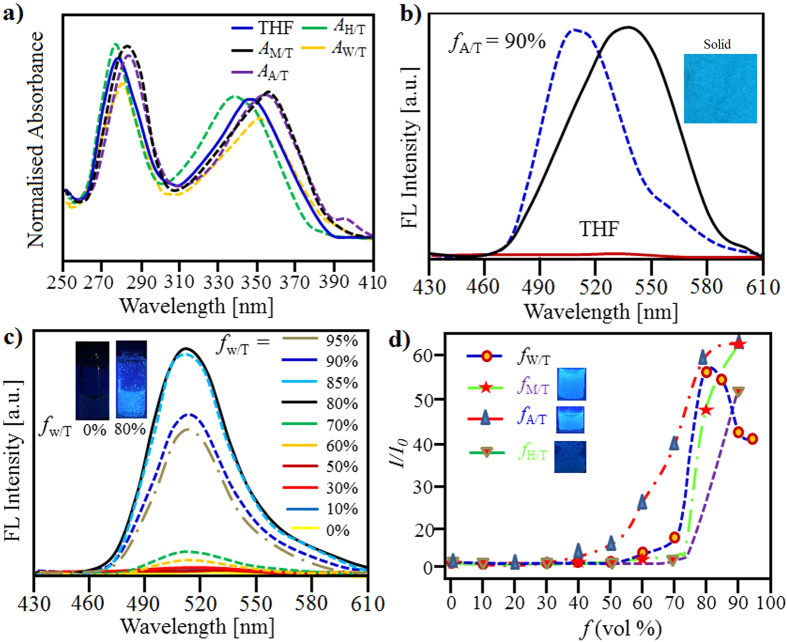
Solution based self-assembly of alkyl-TPE. (**a**) UV-vis absorption spectra in 80% *v/v* of water/THF (*A*_W/T_), acetonitrile/THF (*A*_A/T_), methanol/THF (*A*_M/T_) and hexane/THF (*A*_H/T_). (**b**) The fluorescence (FL) in THF, *A*_A/T_ and solid form. (**c**) FL spectra in *A*_W/T_ mixtures with different *f*_w_ values. (**d**) The changes in Φ_*F*_and λ_em_ with different fractions *f*_W_, *f*_M_, *f*_A/T_ and *f*_H/T_, respectively. The inset of (**b–d**) shows the photographs of the FL images of **alkyl-TPE** (5 μM, λ_ex_ = 365 nm) respective mixture.

The UV-vis absorption of **alkyl-TPE** in tetrahydrofuran (THF) solution showed two well resolved absorption bands with vibrational sub-structure at 273 nm and 343 nm, which is characteristic of the S_0_–S_1_ transition. The s0-S1 transition of **alkyl-TPE** is due to TPE-aromatic molecule with sp^2^-hybridisation of the binding orbitals of its carbon atoms, has a π-π* transition as lowest electronic transition (S0-S1). Figure 3a shows the absorption spectra of **1** in various ratios of mixed solvents such as water/THF (*A*_W/T_), acetonitrile/THF (*A*_A/T_), methanol/THF (*A*_M/T_) and hexane/THF (*A*_H/T_). At concentration of 80% MeOH, ACN and water in THF, the aggregation effects can be clearly seen. Typically, in *A*_W/T_, a reduction in absorption peak intensity is observed. The absorption maximum in *A*_A/T_ and *A*_M/T_ (80%, *v/v*) shows a significant blue-shift of 22 nm (365 nm), respectively, which is typical for J-type aggregates^38^. While, the absorption of **1** in *A*_H/T_ (80%, *v/v*) gives a 16 nm red-shift, similar to H-type aggregates^38^.

The fluorescence (FL) emission spectra of **alkyl-TPE** showed no detectable signals in neat THF upon excitation in the S_0_–S_1_ absorption band at 343 nm (Fig. 3b) and FL quantum yield (Φ_*F*_) is approximately 0.07%. This clearly shows that due to the active intramolecular rotation (IMR) process of phenyl rotors the excited state’s energy is consumed. The FL Φ_*F*_ of the samples with absorption (intensity ~0.05) was estimated using fluorescein in ethanol (Φ_*F*_ = 70%) as standard solution and in solid form was measured using an integrating-sphere photometer. The emission of **alkyl-TPE** in its solid form exhibited strong luminescence with emission peaks at 545 nm (Φ_*F*_ = 14.3%) and red shifted (50 nm) as compared to the aggregated form of **alkyl-TPE** in acetonitrile/THF (*f*_A/T_) i.e. a peak at 495 nm with Φ_*F*_ = 19.7%. The FL spectra of **alkyl-TPE** in water/THF (*f*_W/T_ = 0–95%, *v/v*), clearly shows that **1** is non-emissive even at *f*_W/T_ = 50% (Fig. 3c). The FL increases upon increasing *f*_W/T_ to 60%, and reaches maximum at *f*_W/T_ = 80% with Φ_*F*_ = 16.1%. However, with increasing the volumetric percentage of water beyond 85%, FL decreases which is indicative of larger aggregate formation. Figure 3d illustrate, the Φ_*F*_in methanol/THF (*f*_M/H_ = 90%) and hexane/THF (*f*_H/T_ = 90%) was calculated to be 19.6 and 9.8%, respectively. The fluorescence emission spectroscopy suggests the formation of face-to-face π-stacks of TPE chromophores in a similar manner that is observed in the case of J- and H-aggregates of aromatic chromophores^38^,^39^,^40^.

### Field Emission Scanning Electron Microscopy (FE-SEM)

**Figure 4:**
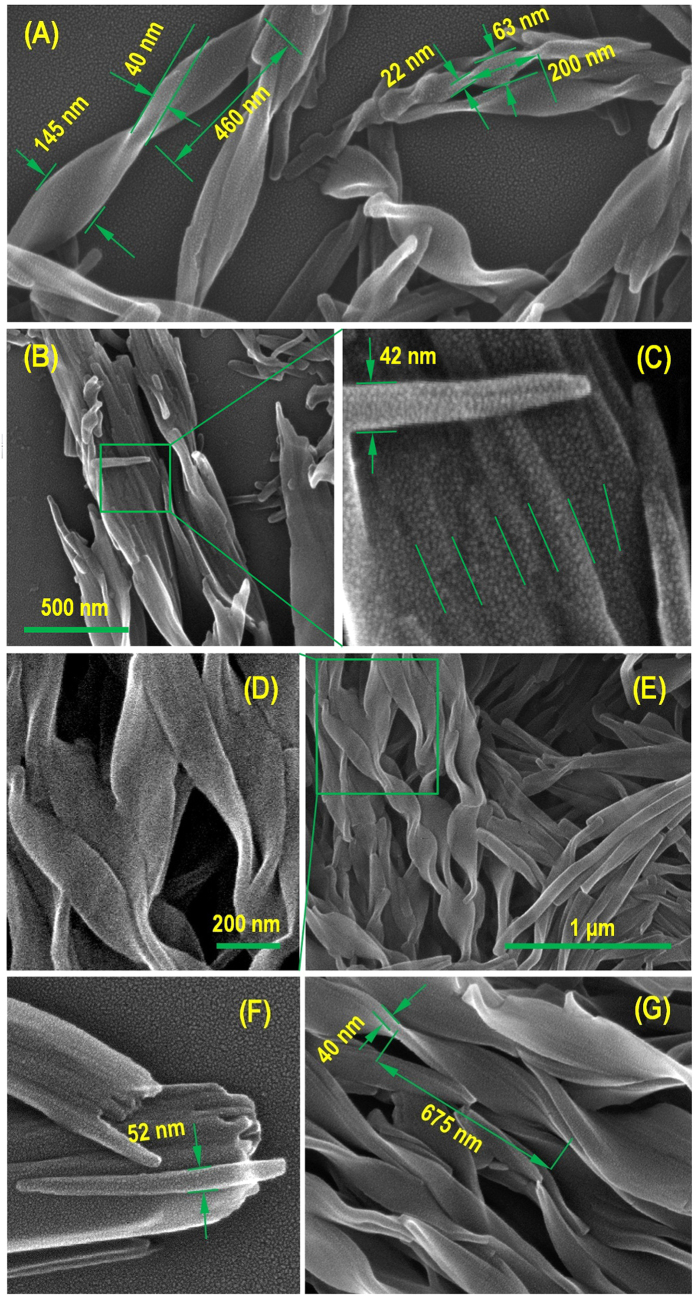
Visualisation of twisted superstructures by SEM analysis. SEM images showing the formation process of the twisted-shaped microstructure in THF/MeOH (**A–C**), and THF/ACN (**D–G**), respectively.

Samples of **alkyl-TPE** were prepared by solvent evaporation on silicon wafer substrate from 1 × 10^−5^ M solution, and SEM studies were performed on samples from THF/hexane (1:9, *v/v*), THF/MeOH (1:9, *v/v*), THF/ACN (1:9, *v/v*) and THF/water (1:7) solvent mixtures (see Fig. 4A–G and ESI Figs 1SA,B and 2SA,B. It is clearly shown in Fig. 4A–C that the self-assembly of **alkyl-TPE** has resulted in highly preferred well-defined right handed twisted ribbons from THF/MeOH and THF/ACN (1:9, *v/v*) solvent mixtures, tens of micrometres in length. The images revealed the long twisted ribbons at the microscopic level, which are approximately 145 nm wide and ~40 nm in thickness with varying twist half period in the range of 460 nm and 675 nm. These supramolecular structures varied in size and dimension, in which the smallest observed feature we could see was a wavy tubular formation ~40 nm in diameter. In some instances these tubular structures hierarchically assemble to form larger ribbon like structures. But the majority of twisted ribbons are composed from stacks of thinner ribbons less than 18 nm in thickness as seen in SEM micrograph in Fig. 4(D,E).

In few instances, the twisted ribbon’s structure energy stability was easily counterbalanced by adhesion forces on the surface where the twist has completely unwound resulting in flat ribbon in contact with silicon wafer surface (see ESI Fig. S3). It is also clear in this image that the mechanical forces applied by the surrounding objects have resulted in disturbing the regularity of the twisted ribbon. This shows that the conformation in the supramolecular arrangement is very sensitive to the mechanical interactions with surrounding environment such as interaction with the substrate surface causing the twist in the ribbon to flatten and produce energetically more stable state. The coexistence of tubular aggregates, thin twisted ribbons, and the superstructures built based on these nanostructures shows the low energy difference between these formations.

### Transmission Electron Microscopy (TEM)

**Figure 5:**
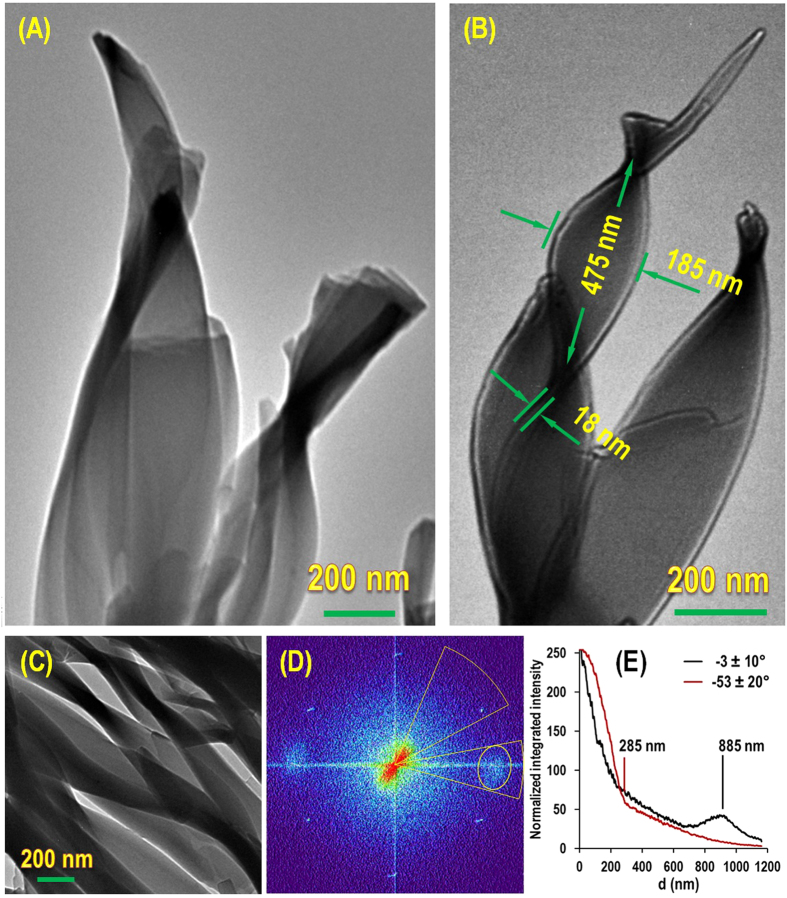
Visualisation of hierarchical supramolecular twisted superstructures by TEM analysis. TEM micrographs of **alkyl-TPE** supramolecular nanostructures formed from (**A**) THF/ACN, and (**B**) THF/MeOH, (**C**) a uniform oriented twisted ribbons in THF/ACN, (**D**) the Fast Furrier Transform (FFT) of image (**C**,**D**) the radial profile of the FFT; in the − 53±10° and −3±20° angles.

To further examine the hierarchical twisted superstructures from the self-assembly of the achiral **alkyl-TPE** molecules in THF/MeOH and THF/ACN solution, the self-assembled chiral aggregates were examined by TEM analysis (Fig. 5), which confirm the formation of right-handed twisted supramolecular assembly. The formation of the twisted ribbons was attributed to the collapsing nanotubes with high diameter and reaching a morphological state with higher thermodynamic stability (Fig. 5A)^41^. Figure 5A clearly show gradual growth of the stacked ribbons on the top of each other. Interestingly, the morphological evolution from nanotubes into twisted ribbons and then stacking thinner ribbons to form multi layered ones were regularly observed (Fig. 5B,C). TEM images revealed the long twisted ribbons at the microscopic level, which measure approximately 185 nm wide and 18 nm thicknesses with a twist half period of approximately 475 nm, similar dimensions ratios are observed in SEM analysis (Fig. 4). Furthermore, image analysis of a uniform samples of the twisted ribbons formed in THF/ACN using Fast Furrier Transform (FFT) revealed a relationship between ribbons width and their twist period, where in this image the width is less than 285 nm and the average half period is 885 nm. Figure 5E shows the radial profile of the FFT of Fig. 5D in the selected angles of 53 ± 10° and −3 ± 20° of the TEM micrograph in Fig. 5C. Image analysis was conducted using Image J 1.49t and radial profile angle plugin to detect the periodicity of the features pattern in the TEM image.

Larger twisted ribbon superstructures were formed in aqueous THF or DMF due to the stronger hydrophobic interactions and solvophobic effects (see ESI Fig. S4A,C), respectively. These larger structures coexist with the previously observed wavy nanotubes in THF/ACN and THF/MeOH with ~40 nm diameter (Fig. 4C,G). From studying the electron microscopy images of the self-assembled **alkyl-TPE** a general idea emerges that initially a wavy tubular structure forms that is able to increase in diameter, these tubular structure >50 nm in diameter collapse to form ribbons with various widths and sizes. This collapse further stabilises the structure by increasing the interaction on the internal wall surface of the nanotubes. These twisted ribbons can further stack to form larger hierarchical superstructure of twisted ribbons.

### Circular dichroism (CD)

**Figure 6:**
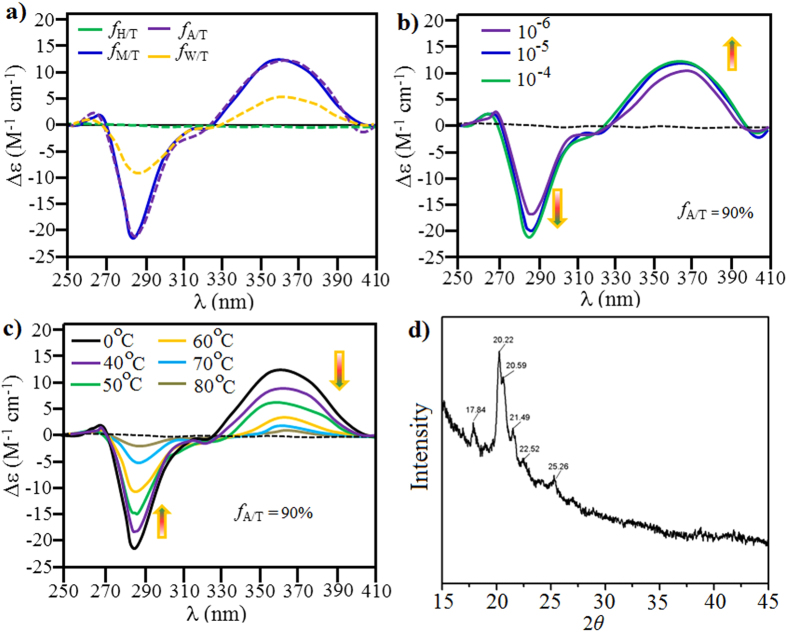
Circular dichroism spectra of alkyl-TPE. (**a**) (**alkyl-TPE** = 10^−5^ M) in hexane/THF (1:9, *v/v*), MeOH/THF (1:9, v/v), ACN/THF (1:9, *v/v*), and water/THF (1:7, *v/v*), (**b**,**c**) in ACN/THF (1:9, *v/v*) with varying concentration and temperature, respectively. (**d**) XRD pattern of the **alkyl-TPE** from ACN/THF mixture (1:9, *v/v)*.

To confirm the handedness of the twisted supramolecular self-assembly in solution, circular dichroism (CD) spectroscopy was performed (Fig. 6)^42^,^43^. The CD was inactive when **1** (10^−5^ M) was dissolved in neat THF or hexane/THF (*f*_*H/T*_ 9:1, *v/v*) solution, as expected for achiral molecules. Intriguingly, the CD spectra of the aggregation show significant Cotton effects through an isodichroic point at the zerocrossing at 321 nm, which is in agreement with the absorption spectrum (Fig. 6a). When **alkyl-TPE** solubilized in 90% v/v *f*_A/T_ or *f*_M/T_, a positive bisignate CD signal was observed in the TPE absorption region, i.e. positive at 362 nm and negative at 286 nm^43^. This is characteristic of excitonically-coupled right handed helical organization of the TPE chromophores^44^. In water/THF (f_W/T_ = 70%) **alkyl-TPE** shows similar right handed chiral CD spectrum, however broadened peaks are observed, which may be due to occurrence of larger aggregates. The CD titration experiments with an increasing concentration of **alkyl-TPE** from 10^−6^ M to 10^−4^ M did not influence the CD spectra. This indicates that **alkyl-TPE** aggregates at concentrations down to 10^−6^ M (Fig. 6b), which is opposite to mixed chiral and achiral molecules where CD activity increases with increasing concentration of chiral molecules^44^. Such efficient chirality induction in self-assembled aggregates of an achiral chromophore was investigated further with temperature dependent CD spectroscopy (Fig. 6c). As expected, with an increase in the temperature above 40 °C, CD spectrum peaks decreased, due to increased solubility of the aggregates to the single molecular level rendering the solution chiral inactive at about 80 °C^45^.

X-ray diffraction (XRD) patterns indicated the crystallinity and the twisted nature of the self-assembly in ACN/THF (1:9, *v/v*) drop casted on silicon wafer. There are peaks at 17.84, 20.22, 20.59, 21.49, 22.52, and 25.26 indicating that the twisted chiral assembly is crystalline in nature. Additionally, the appearance of many peaks in small range of 2*θ* from 17° to 25° is evidence for a typically twisted assembly formation (Fig. 6d)^46^.

TEM and SEM images confirm that these twisted ribbons are indeed formed by right-handed helices twined together. The circular dichrosim also showed the formation of right handed twisted superstructures. Thus, **alkyl-TPE** self-assembles into a supramolecular chiral twisted structure with the hydrophobic chains exposed externally to the polar solvent with the twisted structure being stabilised by intermolecular H-bonding in combination with pi-pi-interaction of AIE-active TPE core.

The **alkyl-TPE** as an amphiphilic molecule in THF/MeOH or THF/ACN solvent mixtures can take conformations where the polar groups of N-H and C = O of the amide group are involved in internal and external H-bonding and the orientation of these H-bonds within the molecule can result in *cis* and *trans* geometries as illustrated in ESI Fig. S5. The *trans* conformation does not possess optical activity due to its centre of symmetry. This is evident in solvents containing hexane where the hydrophobic octyl groups are well solvated. The *cis* conformation where the molecule is polarized is likely to generate the chiral self-assembly with the preferred handedness, resulting in significant structural, mechanical and optical properties.

**Figure 7:**
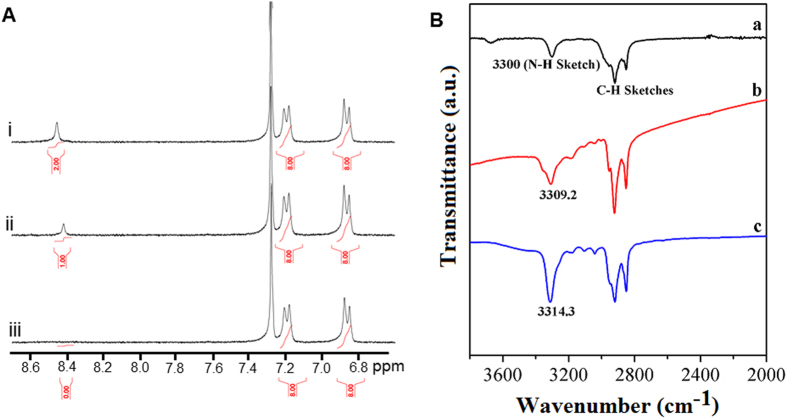
(**A**) ^1^H-NMR spectra of **alkyl-TPE** in the (i) CDCl_3_/MeOD (1:0.2, v/v) (ii) CDCl_3_/MeOD (1:0.4, *v/v*) and (iii) CDCl_3_/MeOD (1:0.6, *v/v*), respectively. (**B**) FT-IR spectra of **alkyl-TPE** by preparing samples on silicon wafer from various solvent mixtures (**a**) THF (**b**) THF/ACN (1:8, *v/v*) and (**c**) THF/MeOH (1:8, *v/v*), respectively.

FTIR spectroscopy and ^1^H NMR were used to evaluate the existence of H-bonding and molecular orientation in the *cis* configuration in the aggregated state (Fig. 7). The ^1^H NMR spectra of **alkyl-TPE** in CDCl_3_/MeOD with varying MeOD fractions showed decreasing N-H amide proton (8.42 ppm) integration with increasing MeOD ratio^47^. These results confirm that the amide N-H is involved in internal and external H-bonding within the aggregated states. Furthermore, FTIR spectra of **alkyl-TPE** shows the expanded amide N-H stretching region (3800–2000 cm^−1^), typically in THF an amide N-H stretching vibration at 3300 cm^−1^, however the peak has broadened and shifted to 3309 cm^−1^ in THF/ACN (1:8, *v/v*) and 3314 cm^−1^ in THF/MeOH (1:8, *v/v*), which correspond to a H-bonded amide N–H stretching band, as reported previously^48^. The band shift in FTIR with 9 and 14 cm^−1^ higher in increasing concentration of acetonitrile and methanol, respectively (ESI Fig. S6), is due to the involvement of amide proton in H-bonding in aggregation states^49^. The free N–H stretching of amide typically occurring near 3440 cm^−1^, was not detected in neither of these solvents. Amide I (1658 cm^−1^) and amide II (1516 cm^−1^), a deformation mode of N-H, in THF/ACN 4 and 6 cm^−1^ and THF/MeOH about 7 and 9 cm^−1^ shift which is higher than that observed in nonbonding amide I and II bands in THF solution, respectively, which adopted a parallel twisted alignment in the crystal. This phenomenon is similar to H-bonding involved in *β*-sheet structures within amyloid-like *β*-sheet assembly^49^. These results clearly show that the amide N–H is mainly involved in both the intermolecular and intramolecular H-bonding with carbonyl of the amide groups. The evidence shown by TEM, SEM, and the positive Cotton effect in CD spectroscopy confirms the right handed chiral nature of the **alkyl-TPE** aggregates^50^.

To explore the TPE molecular design, we also synthesised oligo (ethylene glycol) functionalised TPE i.e. **Oligo-TPE** (see ESI Scheme S1), for which no change was observed in absorption and fluorescence spectroscopy in any proportion of good/poor solvent mixtures such as water/THF, acetonitrile/THF, methanol/THF and hexane/THF. Also no self-assembled supramolecular aggregation was observed using SEM microscopy in any proportion of the above solvents (ESI Fig. S7).

## Discussion

Supramolecular hierarchical twisted ribbons were observed in several self-assemblies of molecules bearing chiral moieties, however, the formation of twisted ribbons from achiral compounds is exceptional. Here we described the formation of chiral twisted superstructures of AIE-active achiral molecule controlled by the solvophobic effect alone. These supramolecular structures varied in size and dimension, where the smallest observed feature we can see is a wavy tubular formation. These tubular structures hierarchically assemble to form the larger ribbon like structures due to the solvophobic interactions. Analyses of TEM and SEM images clearly show that these twisted ribbons are indeed formed by right-handed helices twined.

Based on above SEM and TEM images, we believe, due to presence of twisted TPE groups along with four long alkyl chains, some change in configuration may occur at the interface of polar and partially polar solvent. Polar solvents induce polarity in the **alkyl-TPE** molecular structure and enhance the hydrophobic interactions between molecules resulting in the twisted chiral self-assembly. The tubular and the twisted ribbon supramolecular structures berry the alkyl chains on the internal side of the wall while exposing the amide groups to form hydrogen bonding with polar solvent on the exterior of the structure. Nonpolar solvents such as hexane and less polar solvents such as THF will strongly solubilise the alkyl chains preventing self-assembly and preferring a more symmetrical conformation. It is clear that solvophobic effects play an important role in supramolecular chiral assembly from this achiral molecule.

We believe conformation of **alkyl-TPE** in solution is influenced by solvent polarity resulting in *cis* and *trans* conformations. The more stable *trans* conformation is more soluble in nonpolar solvents and is internally stabilized in comparison to the *cis* conformation. However, the *cis* conformation is more prone to stabilize *via* intramolecular interactions in self-assembly and solvation in relatively polar solvents. The **alkyl-TPE** as an amphiphilic molecule in THF/MeOH or THF/ACN solvent mixtures can take conformations where the polar groups of N-H are involved in internal and external H-bonding and the orientation of these H-bonds within the molecule can result in two geometries as seen in *cis* and *trans* for details see ESI Fig. S5. The *trans* conformation does not possess optical activity due to its centre of symmetry. This is evident in solvents containing hexane where the hydrophobic octyl groups are well solvated. The *cis* formation where the polar groups are on one side and the nonpolar hydrophobic alkyls on the other are the likely conformation in the chiral self-assembly with the preferred handedness, resulting in significant structural, mechanical and optical properties. ^1^H NMR and FTIR spectroscopy was further used to determine the role of H-bonding interaction and molecular orientation in *cis* configuration in the aggregated state (Fig. 7).

The present controlled supramolecular chiral system is particularly interesting, since the present system is based on AIE-active component and may offer practical value; for example, templates for helical crystallisation and development of mechanochromic luminescent materials for optoelectronic devices.

## Materials and Methods

### Material and measurements

Tetraphenylethene, chloroform (CHCl_3_), chloroform-d (CDCl_3_), methanol (MeOH), dichloromethane (DCM), tetrhedrofuran (THF), and *N,N’*-dimethylformamide (DMF) were purchased from Aldrich and used without purification, unless otherwise specified. ^1^H NMR, ^13^C-NMR spectra were recorded on a Bruker spectrometer using CDCl_3_ and MeOD as solvent and tetramethylsilane as an internal standard. The solvents for spectroscopic studies were of spectroscopic grade and used as received. Mass spectra (MS) were obtained using Bruker AutoFlex Matrix Assisted Laser Desorption/Ionisation—Time of Flight—Mass Spectrometer (MALDI-TOF-MS). The X-ray diffraction (XRD) analysis was performed using a Bruker D8 FOCUS diffractometer with a Cu target radiation source (λ = 0.15418 nm).

### Spectroscopic measurements

#### UV-vis measurements

UV-vis absorption spectra were recorded in a Cary 50 spectrometer in 1cm path length cuvette. A 0.2 mL aliquot of 10^−3^ M stock solution of **alkyl-TPE** was transferred to various ratios of water/THF, ACN/THF, Hexane/THF and methanol/THF in different volumetric flasks, and made up to 2 mL volume. The solutions were allowed to equilibrate for 2 h prior to the spectroscopic measurements.

#### Fluorescence Measurements

Fluorescence emission spectra were recorded in a Horiba Jobin Yvon FluoroMax®-4–Spectrofluorometer. Fluorescence measurements and quenching experiments were performed on a FluoroMax-4 equipped with an injector port and stirrer temperature controlled at 25 °C. All experiments were performed in a quartz cell with a 1 cm path length (λ_ex_ = 365 nm).

#### Scanning Electron Microscopy (SEM) imagining

The silicon wafer substrates were cleaned by rinsing in acetone, ethanol and then Milli Q water. SEM Samples were prepared by solvent evaporation on these substrates and then sputter coated with gold for 10 s at 0.016 mA Ar plasma (SPI, West Chester, USA) for SEM imaging using a FEI Nova NanoSEM (Hillsboro, USA) operating at high vacuum which provided direct visualisation of the self-assembled.

#### Transmission Electron Microscopy (TEM) imagining

TEM samples were prepared by solvent evaporation on a holey carbon grid and micrographs were produced using a Jole 1010 100 kV TEM.

## Additional Information

**How to cite this article**: Anuradha *et al*. Right handed chiral superstructures from achiral molecules: self-assembly with a twist. *Sci. Rep*. **5**, 15652; doi: 10.1038/srep15652 (2015).

## Supplementary Material

Supplementary Information
