# Supplementary material for: Right handed chiral superstructures from achiral molecules: self-assembly with a twist
Source: Sci Rep. 2015 Oct 23;5:15652. doi: 10.1038/srep15652 (PMC4616054; doi:10.1038/srep15652)
Supplement: Supplementary Information [file srep15652-s1.doc]

**Supplementary information**

**Right handed chiral superstructures from achiral molecules: self-assembly with a twist**

Anuradha, La Duc Duong, Mohammad Al Kobaisi, Sheshanath V. Bhosale*

School of Applied Sciences, RMIT University, GPO Box 2476, Melbourne, Vic. 3001, Australia

Corresponding authors: Tel.:+61399252680; E-mail: sheshanath.bhosale@rmit.edu.au


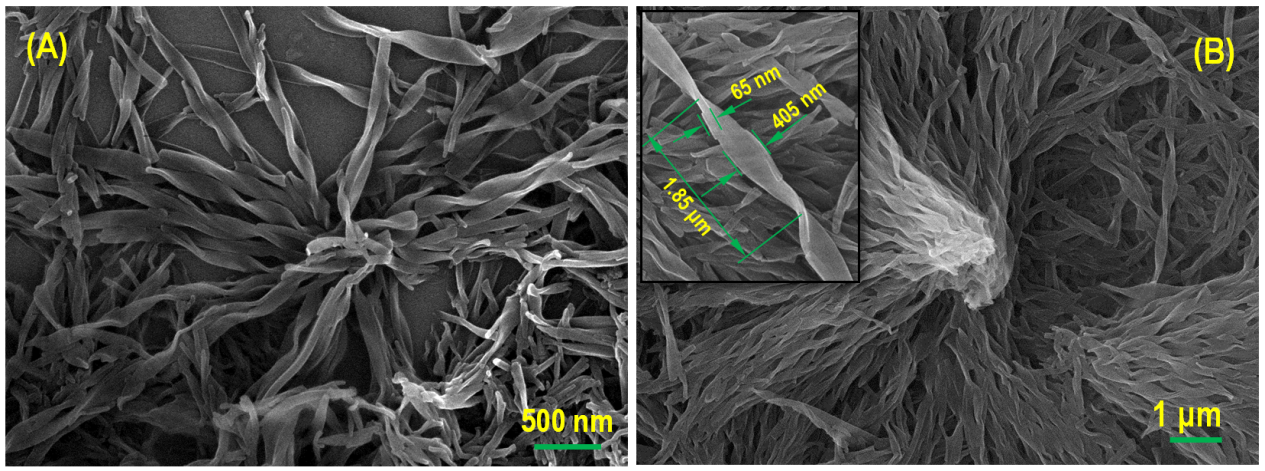


**Figure S1 |** SEM micrographs of **alkyl-TPE** supramolecular self-assembly in (A) THF/MeOH, (B) THF/ACN,


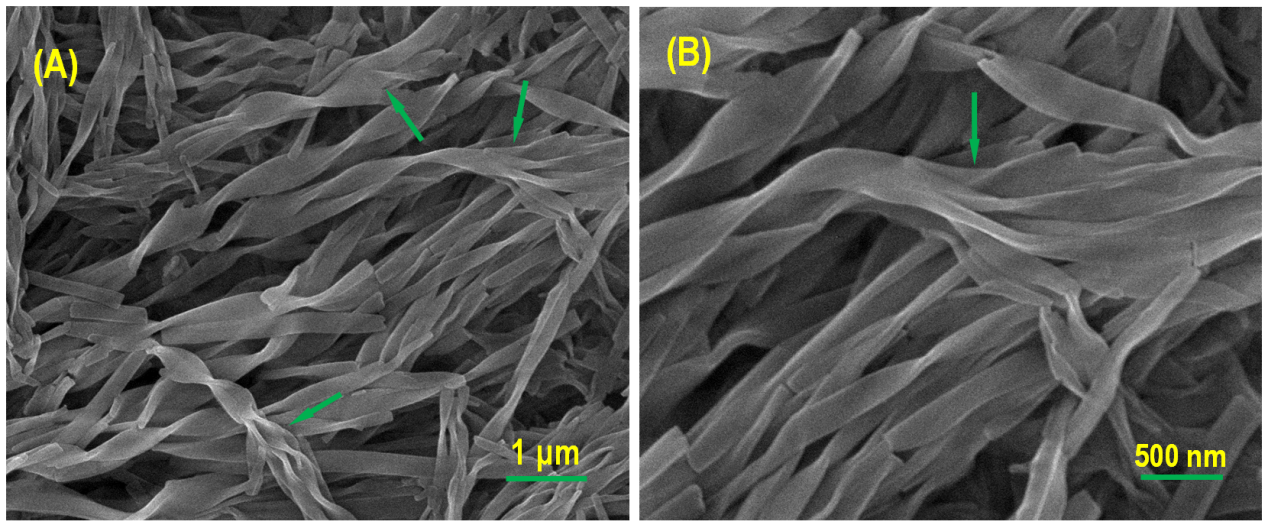


**Figure S2 |** SEM micrographs of **alkyl-TPE** supramolecular self-assembly where thinner ribbons stacking to form thicker ones in THF/ACN (A), and (B) a selected enlarged area where this is clearly shown.


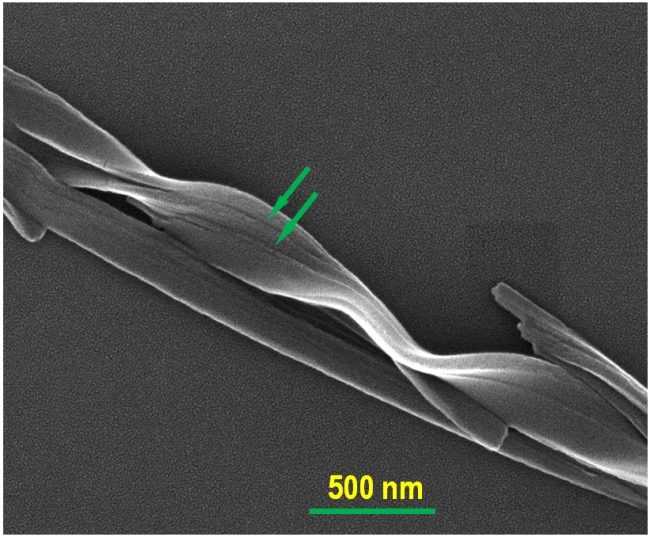


**Figure S3 |** **Visualisation of twisted superstructures by SEM analysis**. SEM micrographs of **alkyl-TPE** supramolecular self-assembly in THF/ACN after solvent evaporation on silicon wafer surface.


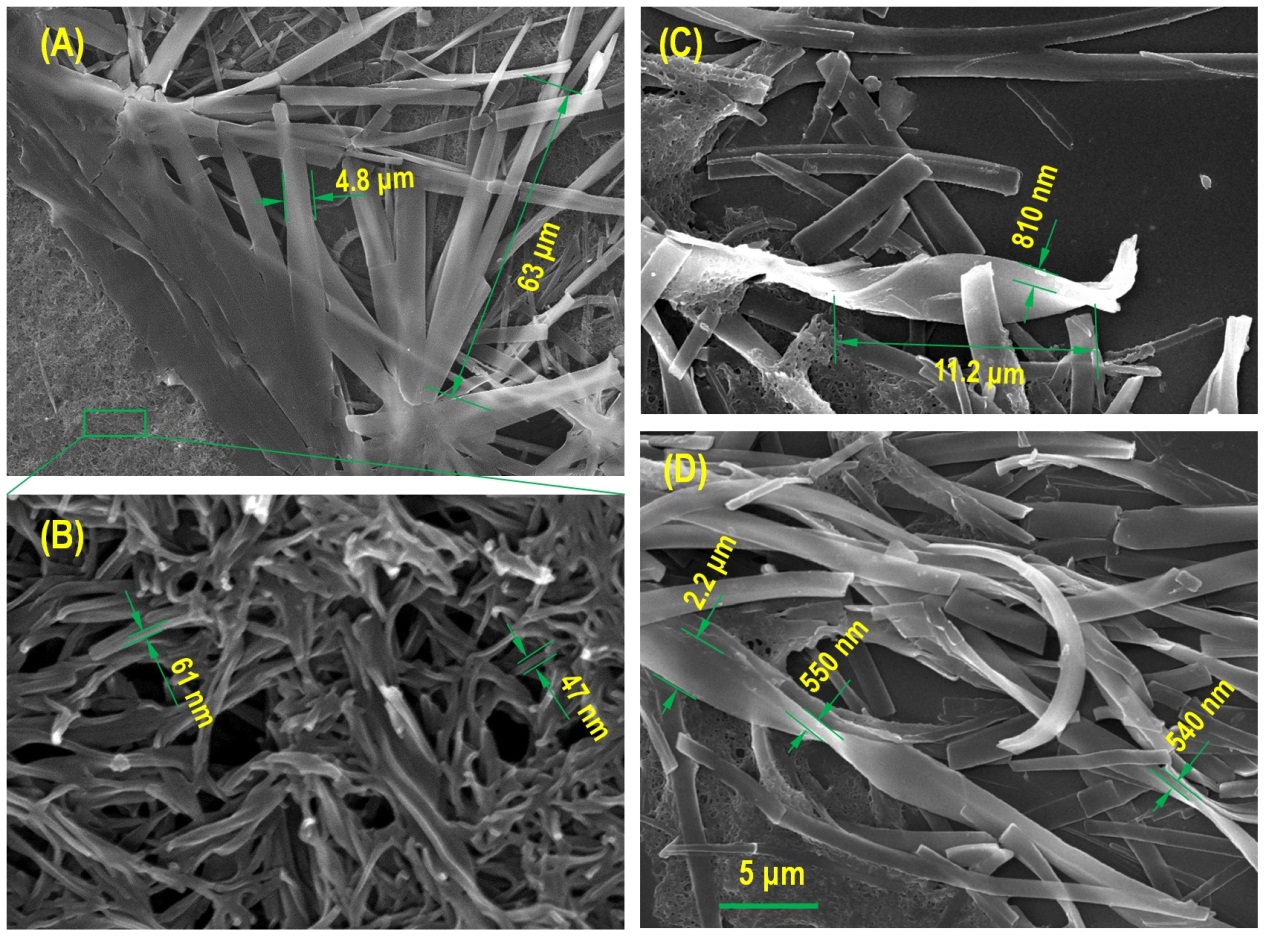


**Figure S4 |** SEM micrographs of **alkyl-TPE** supramolecular self-assembly in (A, B) DMF/water, and in (C, D) THF/water.

*trans cis*

**Figure S5 |** Schematics of **alkyl-TPE** amphiphilic molecule in two geometries *cis* and *trans* through internal and external H-bonding and the orientation of these H-bonds within the molecule.


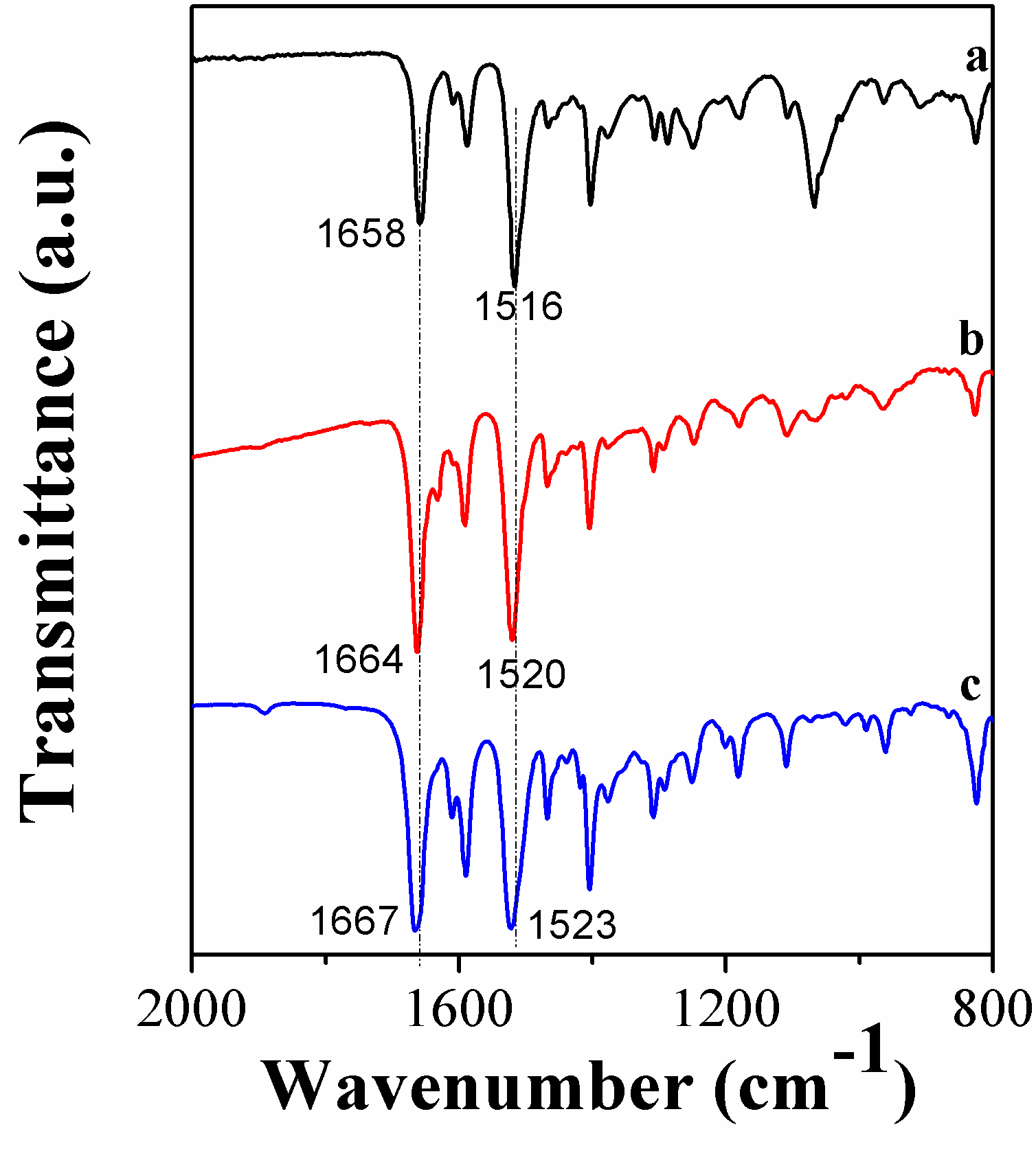


**Figure S6 |** FTIR spectra of **alkyl-TPE** by preparing samples on silicon wafer from various solvent mixtures (a) THF (b) THF/ACN (1:8, *v/v*) and (c) THF/MeOH (1:8, *v/v*), respectively.


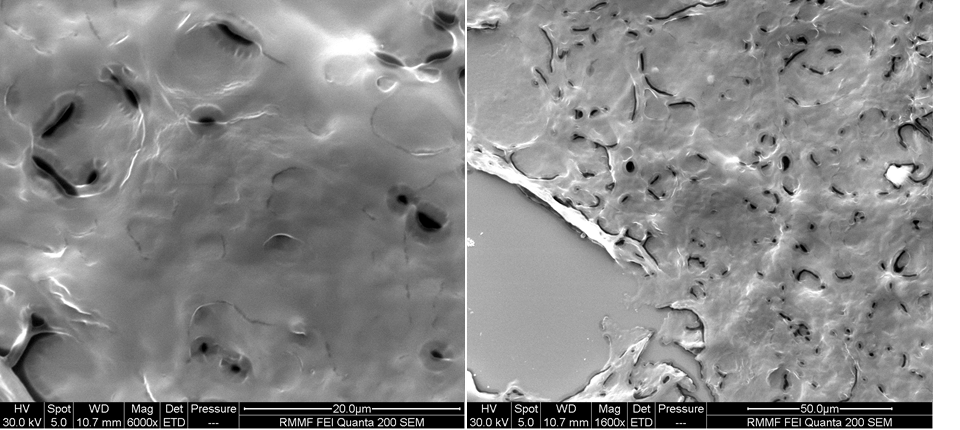


**Figure S7 |** SEM micrographs of oligo-TPE in 1/9, *v/v* THF/ACN (left) and THF/methanol (right), respectively.


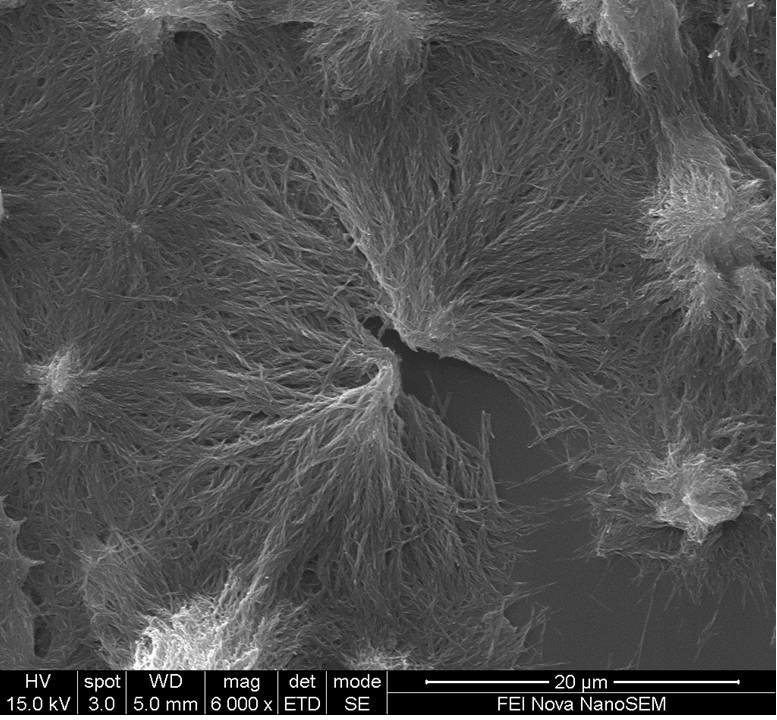


**Figure S8 |** SEM micrographs of **alkyl-TPE** (10^-4^ M) in THF/ACN (1:9, *v/v*).


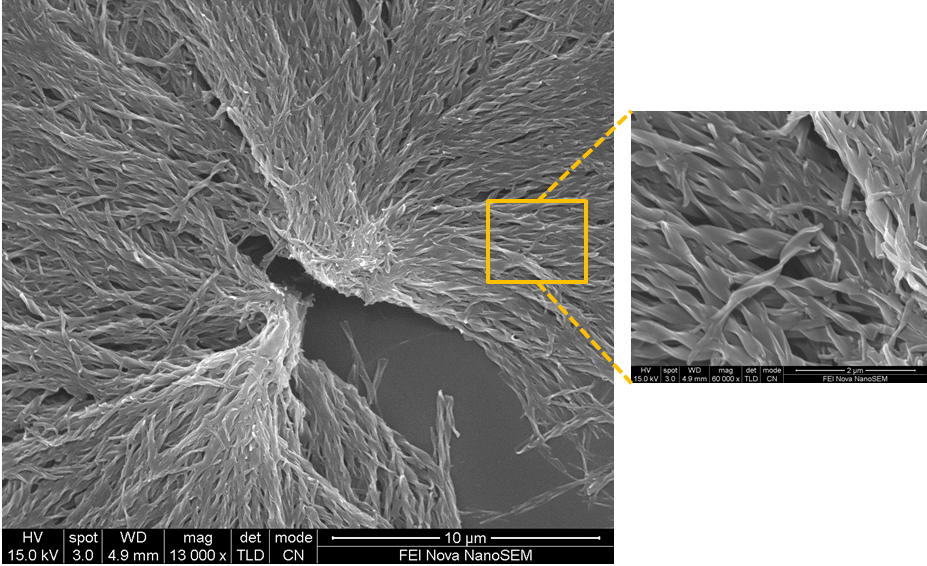


**Figure S9 |** Enlarge SEM micrographs **Extended Figure S8**.


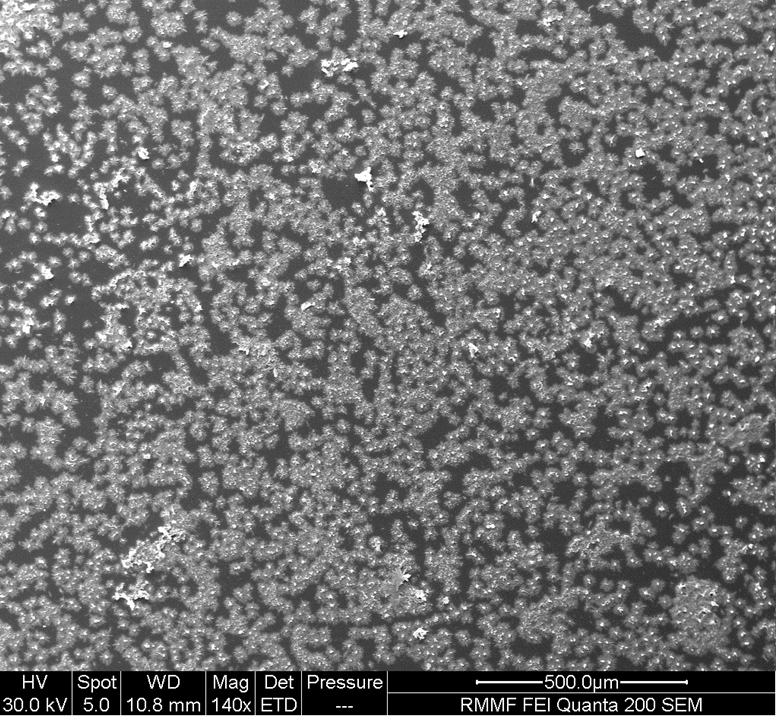


**Figure S10 |** SEM micrographs of **alkyl-TPE** (10^-4^ M) in THF/methanol (1:9, v/v).


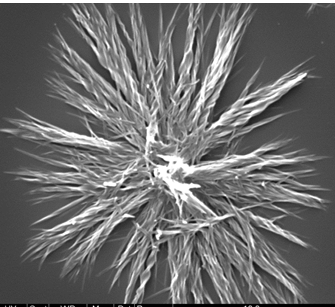


**Figure S11 |** Enlarge SEM micrographs of **Extended Data Figure S10**.


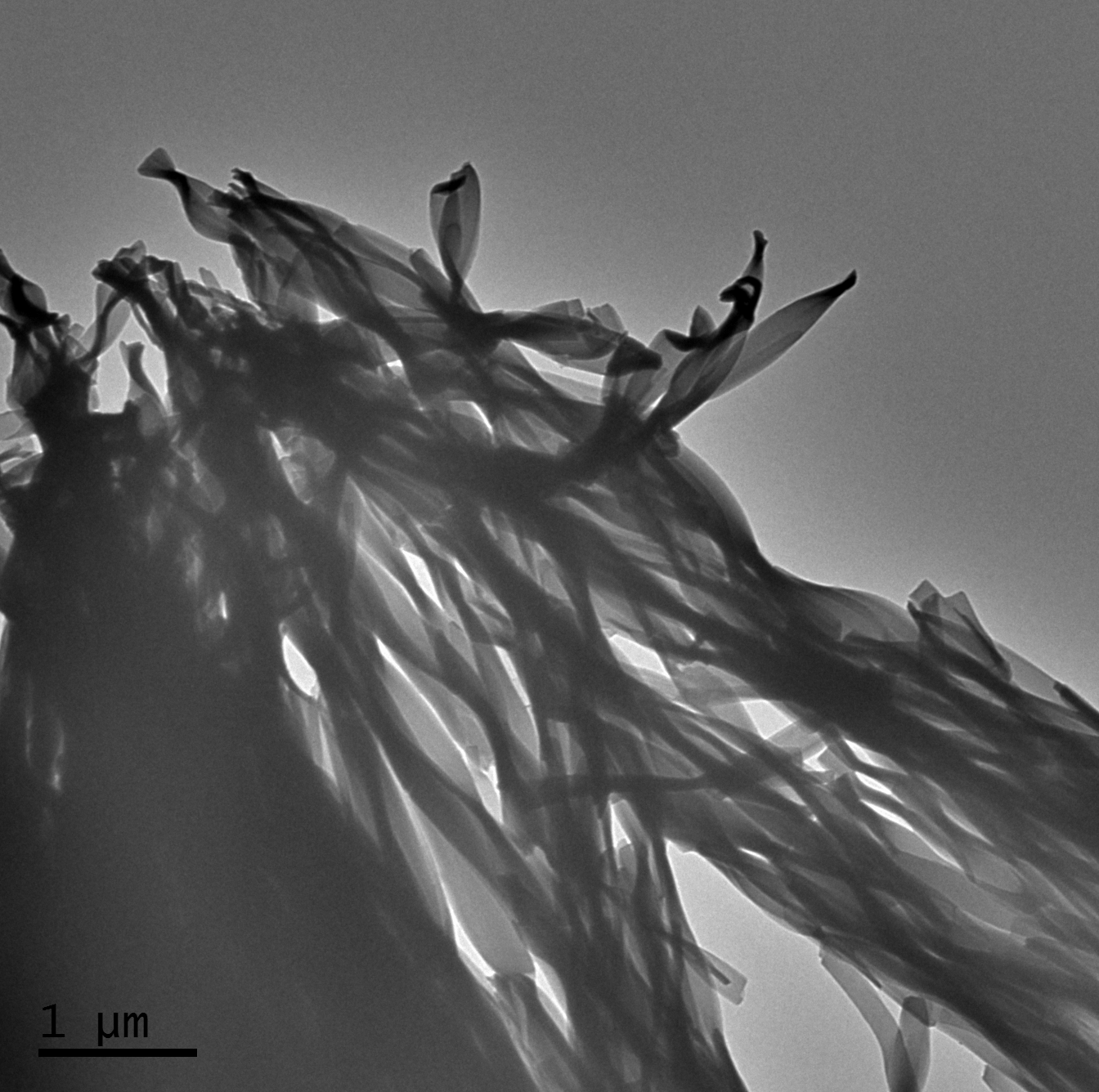

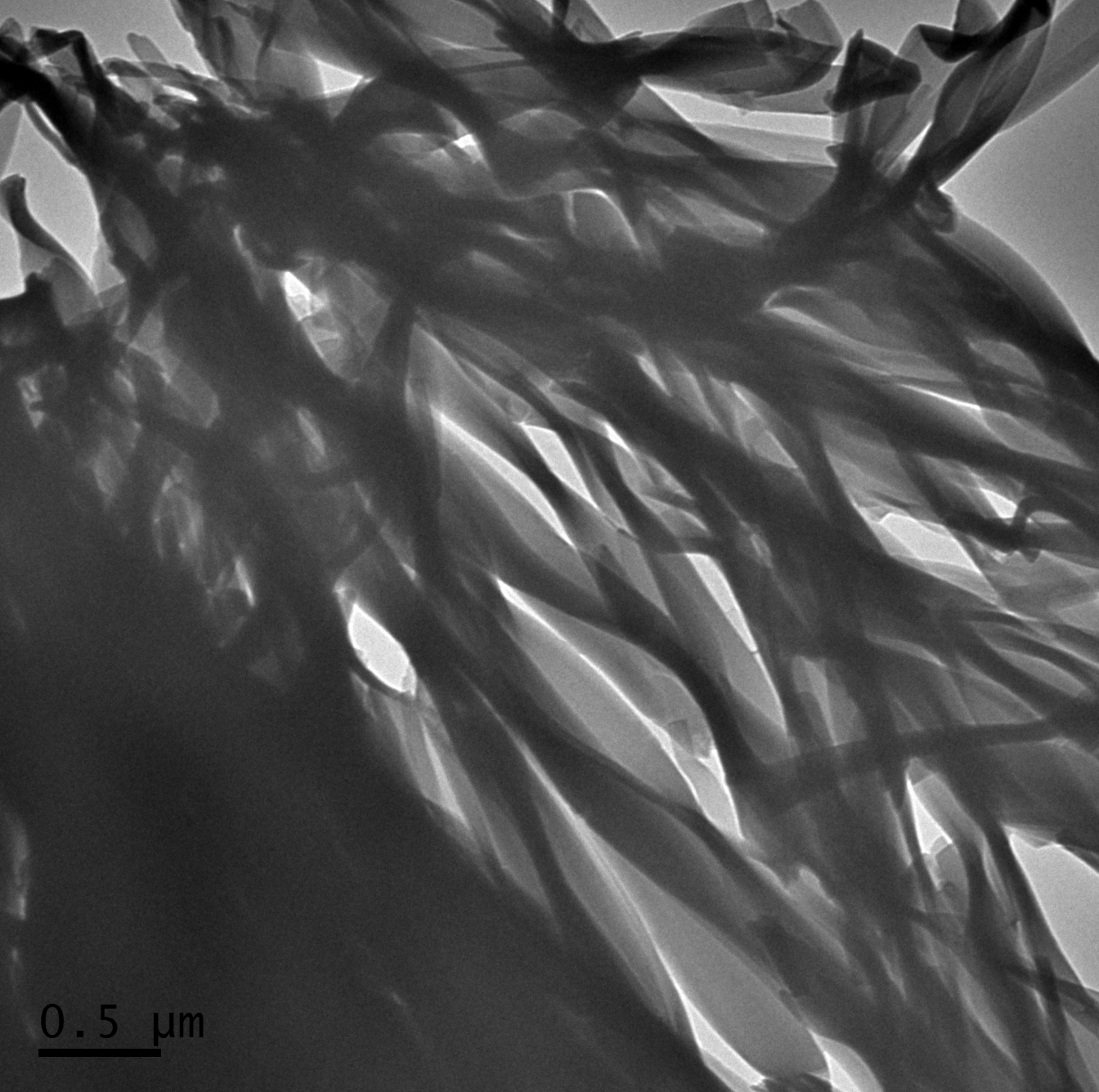


**(A)**

**(B)**


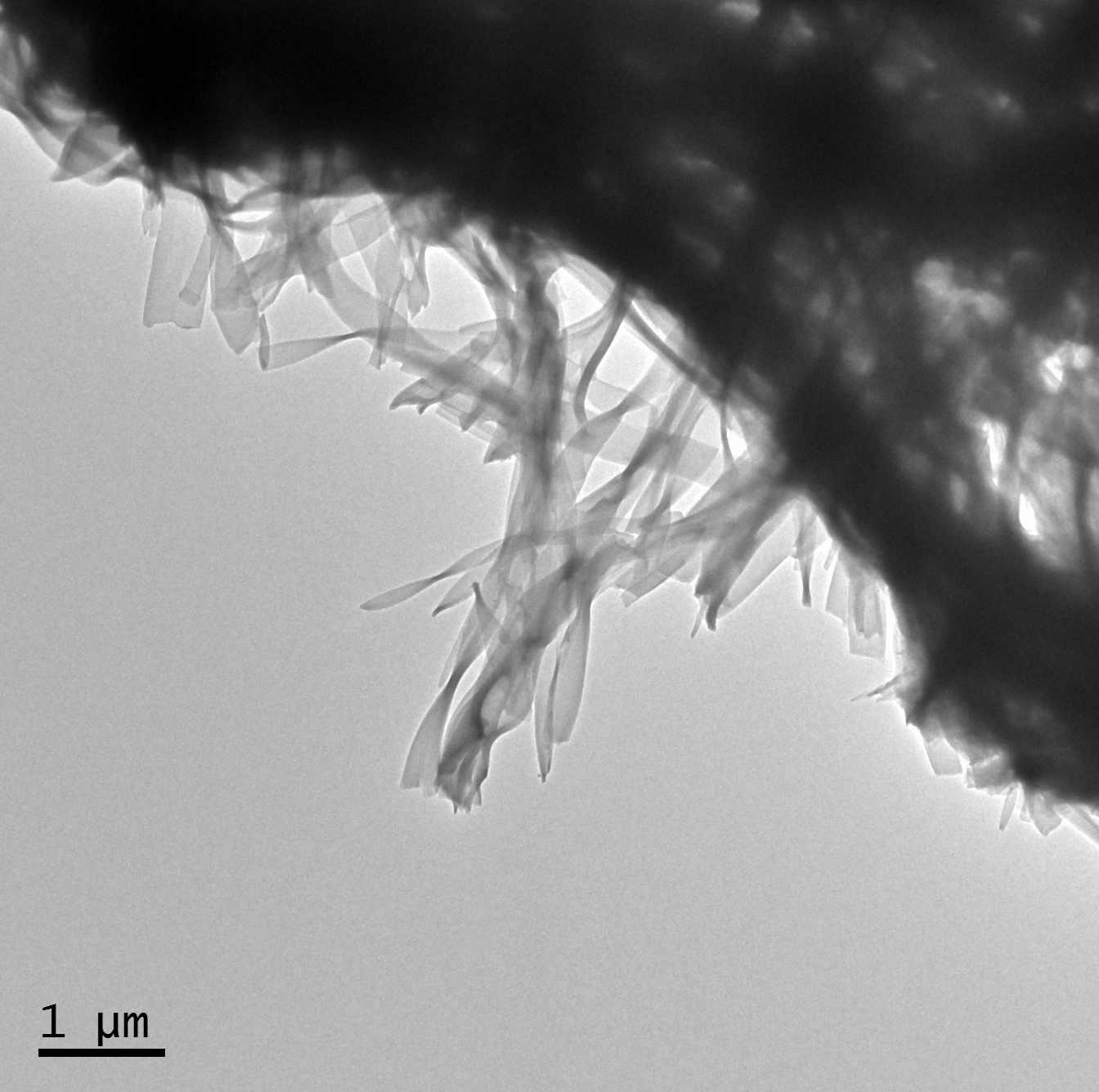

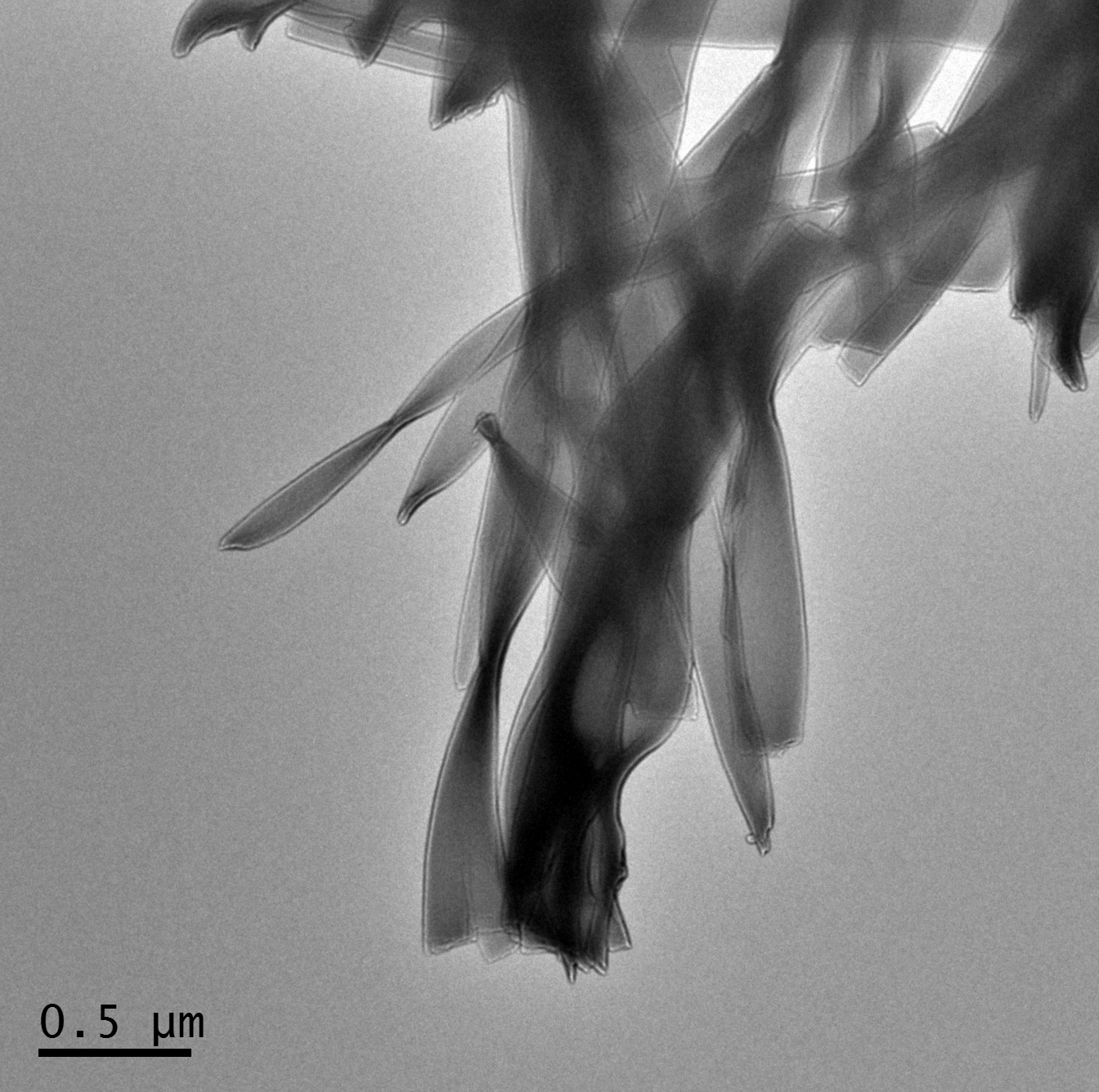


**(C)**

**(D)**

**Figure S12 |** TEM micrographs of **alkyl-TPE** supramolecular self-assembly in (A and B) THF/ACN, and (C and D) THF/ACN.

Experimental procedures

Material and methods

**Figure S12 |** Synthesis of structures used for self-assembly studies.

**Step-1:** Synthesis of 1, 1, 2, 2-tetrakis (4-nitrophenyl) ethene (**1**): To a stirred solution of tetraphenylethylene (2 g, 6.01 mmol) in DCM (60 mL) was added Conc. H_2_SO_4_ (4 mL) and fuming HNO_3_ (15 mL) and resultant reaction mixture was stirred at RT for 16 h. Reaction completion was checked by TLC analysis. After completion, reaction was quenched with water and extracted with CHCl_3_ (3 x 30 mL). The combined organic layer was dried over anhydrous MgSO_4_ and evaporated on rota vapour to complete dryness. Crude residue obtained was purified by flash column chromatography to afford 1 as a yellow solid (2.5 g, yield 81.2%). ^1^H NMR (300 MHz, CDCl_3_): δ 8.09 (d, *J* = 9 Hz, 8H), 7.19 (d, *J* = 9 Hz, 8H); ^13^C NMR (75 MHZ, CDCl_3_): δ 147.4, 147.1, 141.6, 131.8, 123.9; MALDI-TOF, m/z: [M^+^] calcd for C_26_H_16_N_4_O_8_: 512.10, found: 512.03.

**Step-2:** Synthesis of *4,4’,4’,4’’’*-(ethene-1, 1, 2, 2-tetrayl) tetraaniline (**2**): To a stirred solution of compound 1 (3.5 g, 6.83 mmol) in EtOH (600 mL) was added Pd/C (0.350 g, cat.) and NH_2_NH_2_.H_2_O (60 mL) in drop wise manner. Resultant reaction mixture was refluxed under intense stirring for 24 h. Reaction progress was checked by TLC analysis. After completion, reaction mixture was allowed to cool to room temperature. Reaction mixture was filtered through celite bed. Celite bed was washed with excess of EtOH. Filtrate was evaporated to complete dryness to give 2 as yellow solid (2.1 g, yield 78.6%). ^1^H NMR (300 MHz, Acetone): δ 6.79–6.68 (m, 8H), 6.44–6.34 (m, 23H), 4.43 (s, 23H); ^13^C NMR (75 MH_Z_, Acetone): δ 206.24, 146.91, 138.39, 135.13, 133.07, 114.36, 30.64, 30.38, 30.13, 29.87, 29.61, 29.36, 29.10; MALDI-TOF, (m/z): [M^+^] calcd for C_26_H_24_N_4_: 392.20, found: 392.20.

Synthesis of *N,N',N'',N'''*-(ethene-1,1,2,2-tetrayltetrakis(benzene-4,1-diyl))tetrakis(decanamide) **4**: To a stirred solution of **3** (0.52 g, 3.05 mmol) in anhydrous DMF (10 mL) were added EDC.HCl (0.47 g, 3.05 mmol) and DMAP (0.37 g, 3.05 mmol) and resultant was stirred at RT for 15 min. Then **2** (0.2 g, 0.51 mmol) was added stirring was continued at RT for 16 h under inert atmosphere. Progress of reaction was monitored by TLC analysis. After completion, reaction was quenched with water, precipitated solid was filtered through Buchner funnel and washed with methanol and dried under vacuum to afford **4** as greyish white solid (0.28 g, yield 55 %). ^1^H NMR (300 MHz,CDCl_3_/MeOD [10/1, v/v]): δ 7.26 (d, *J* = 8.5 Hz, 8H), 6.90 (d, *J* = 8.5 Hz, 8H), 2.27 (t, *J* = 7.6 Hz, 8H), 1.62 (t, *J* = 7.4 Hz, 8H), 1.27 (m, 56H), 0.82 (t, *J* = 6.4 Hz, 12H); ^13^CNMR (75 MHz, CDCl_3_/MeOD [10/1, v/v]): δ 172.8, 139.2, 136.3, 131.4, 118.8, 36.8, 31.4, 29.0, 29, 28.9, 28.8, 25.4, 22.2, 13.5; MALDI-TOF: [M^+^] calcd for C_66_H_96_N_4_O_4_: 1008.74 (M)^+^, found: 1008.82 (M)^+^, 1031.82 (M+Na)^+^ and 1047.80 (M+K)^+^.

Synthesis of N,N',N'',N'''-(ethene-1,1,2,2-tetrayltetrakis(benzene-4,1-diyl))tetrakis(2-(2-(2-methoxyethoxy)ethoxy)acetamide **6**: EDC.HCl (0.76 g, 4.88 mmol), DIPEA (1.77 mL, 8.79 mmol) and HOBt (0.75 g, 4.89 mmol) were added to a solution of **2** (0.2 g, 0.509 mmol) and 5 (0.72, 4.04 mmol) in dry DMF (10 mL). The reaction mixture was stirred at RT for 16 h. Reaction progress was monitored by TLC. After completion, reaction was quenched with water and extracted with chloroform (3x50 mL). Organic layer was separated and washed with sat. NaHCO_3_ followed by 2M HCl. Dried over MgSO_4_ and evaporated under reduced pressure to give crude residue which was further purified by flash column chromatography to afford **6** as yellow solid (0.316 g, yield 60%). ^1^H NMR (300 MHz, CDCl_3_) δ 8.64 (s, 4H), 7.36 (d, *J* = 8.6 Hz, 8H), 6.98 (d, *J* = 8.6 Hz, 8H), 4.06 (s, 8H), 3.76–3.66 (m, 24H), 3.54–3.51 (m, 8H), 3.29 (s, 12H); ^13^C NMR (75 MHz, CDCl_3_) δ (ppm): 168.0, 139.9, 139.6, 1135.8, 132.1, 119.2, 71.9, 71.2, 70.8, 70.5, 70.2, 59.0; MALDI-TOF: [M] ^+^ calcd for C_54_H_72_N_4_O_16_: 1032.494 (M)^+^, found: 1032.567 (M)^+^, 1055.533 (M+Na)^+^ and 1071.514 (M+K)^+^.


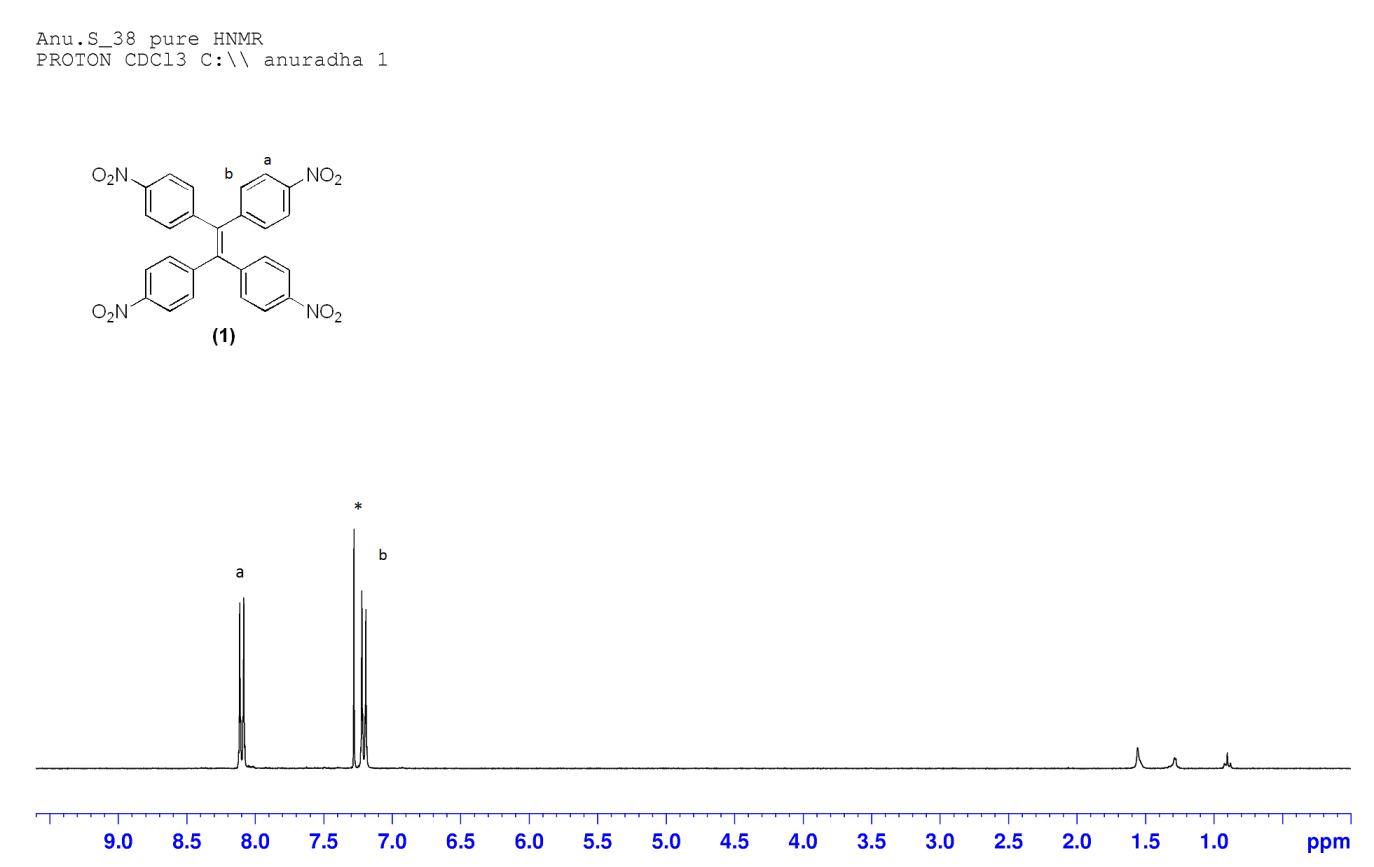


^1^HNMR of 1,1,2,2-tetrakis(4-nitrophenyl) ethene (**1**)


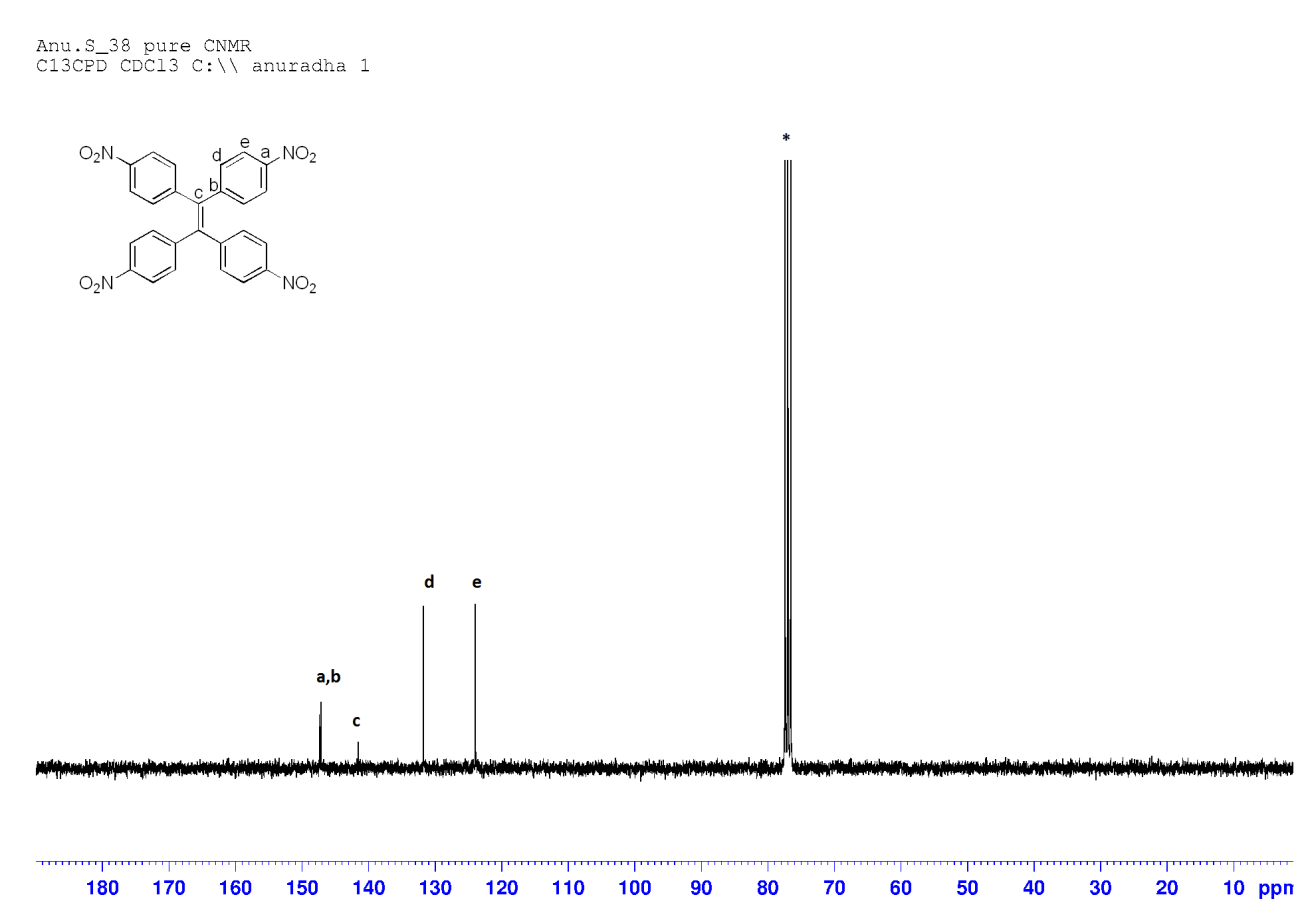


^13^CNMR 1,1,2,2-tetrakis(4-nitrophenyl) ethene (**1**)

**
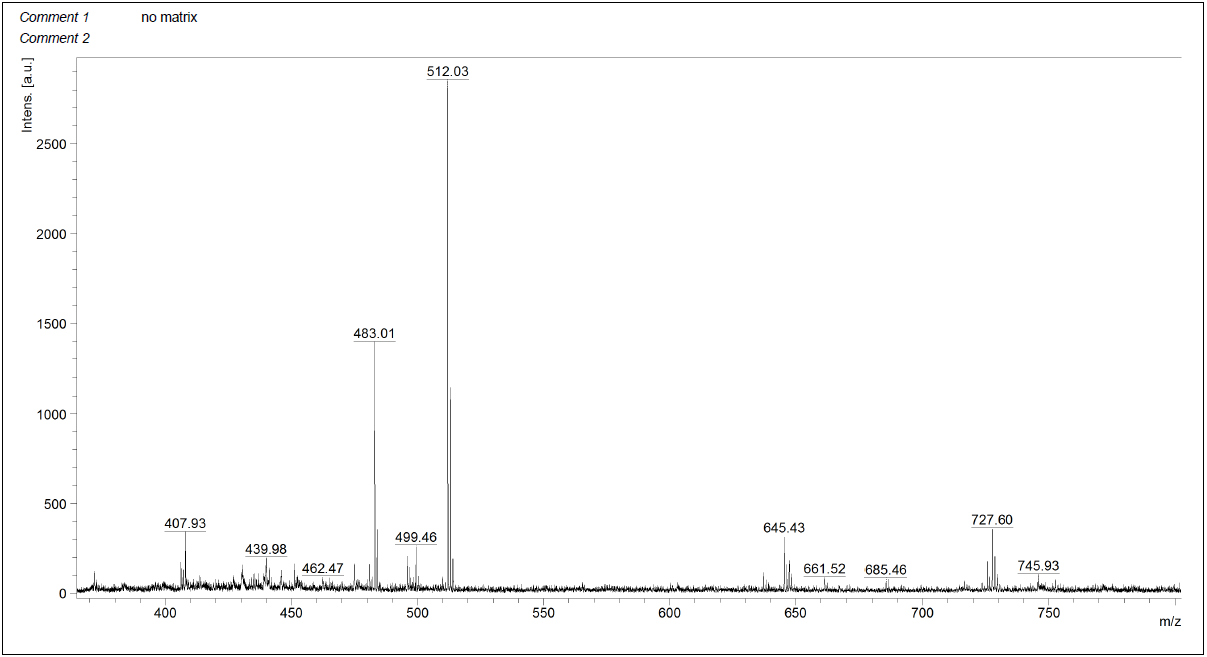
**

MALDI-TOF 1,1,2,2-tetrakis(4-nitrophenyl) ethene (**1**)


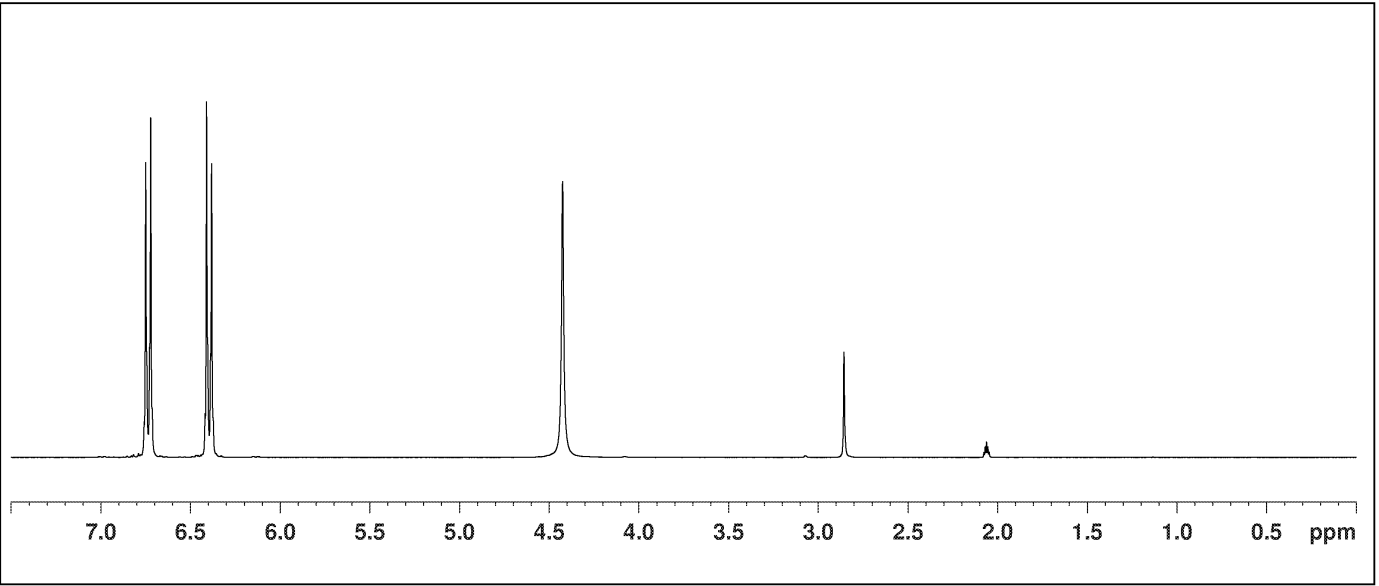


^1^HNMR of *4,4’,4’,4’’’*-(ethene-1, 1, 2, 2-tetrayl) tetraaniline (**2**)

**
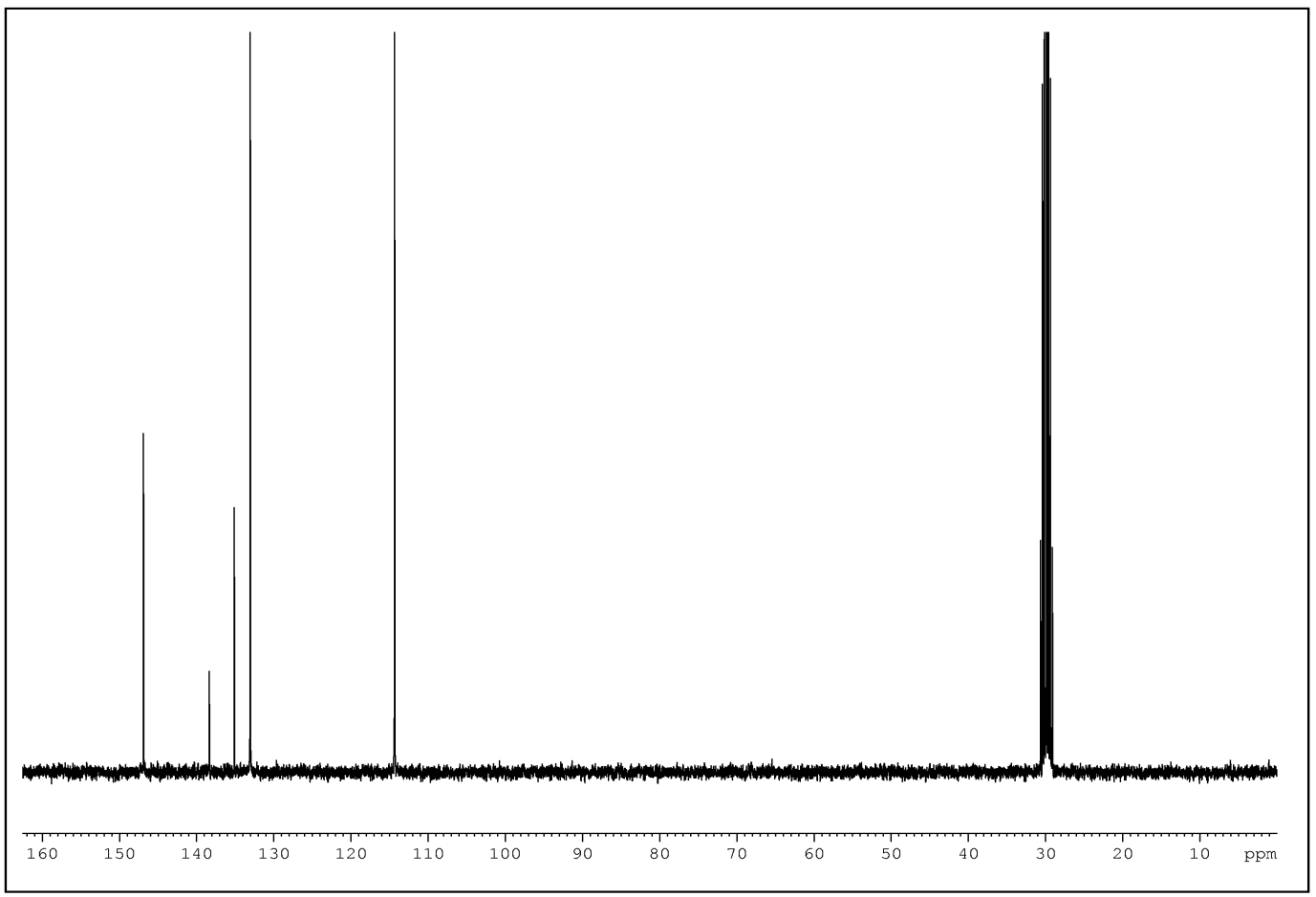
**

^13^CNMR of *4,4’,4’,4’’’*-(ethene-1, 1, 2, 2-tetrayl) tetraaniline (**2**)


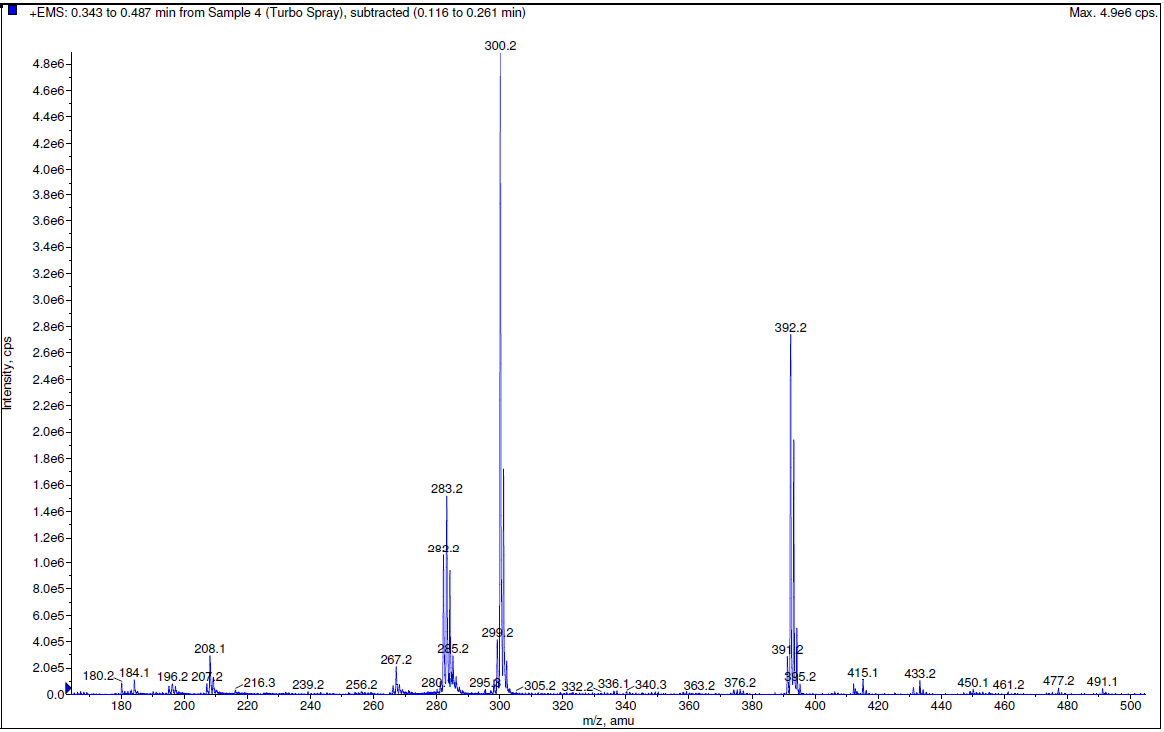


MALDI-TOF of *4,4’,4’,4’’’*-(ethene-1, 1, 2, 2-tetrayl) tetraaniline (**2**)


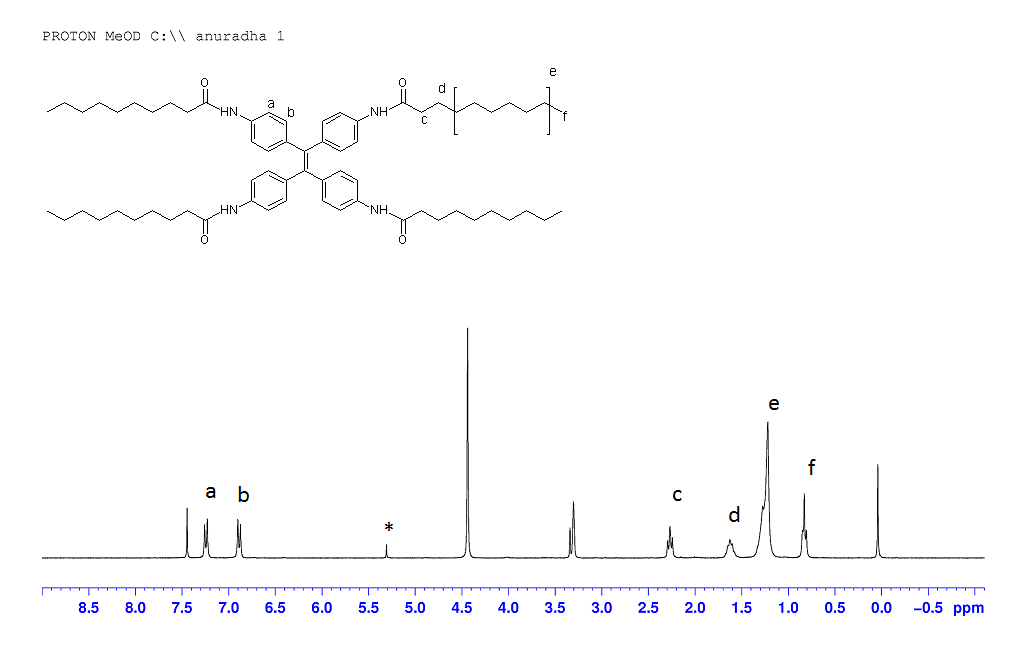


^1^HNMR *N,N',N'',N'''*-(ethene-1,1,2,2-tetrayltetrakis(benzene-4,1-diyl))tetrakis(decanamide) **4**

**
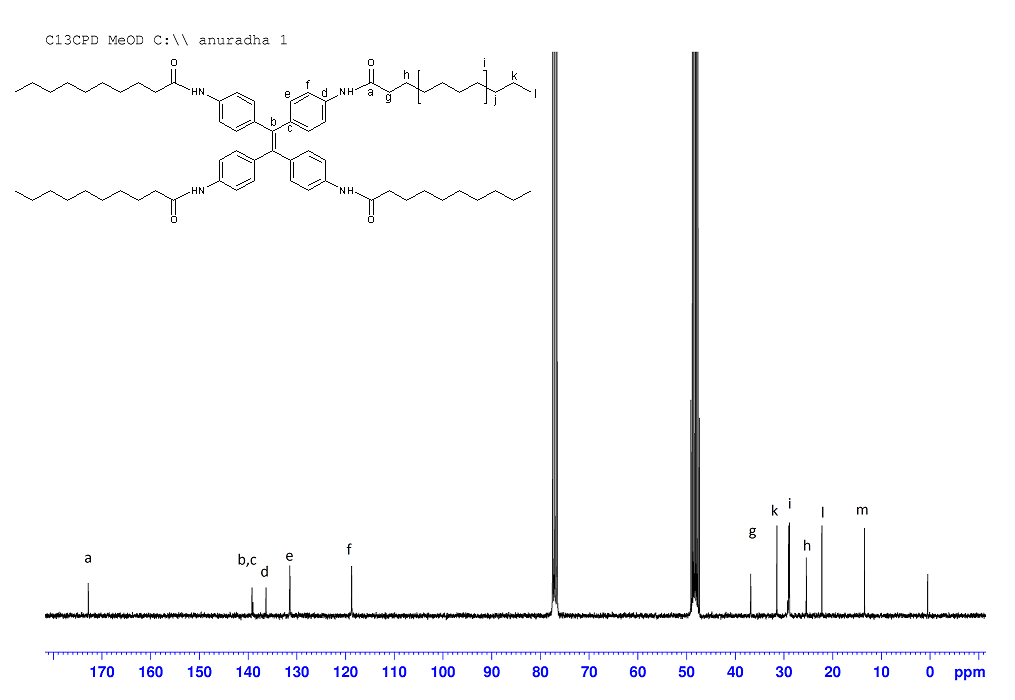
**

^13^C NMR *N,N',N'',N'''*-(ethene-1,1,2,2-tetrayltetrakis(benzene-4,1-diyl))tetrakis(decanamide) **4**

**
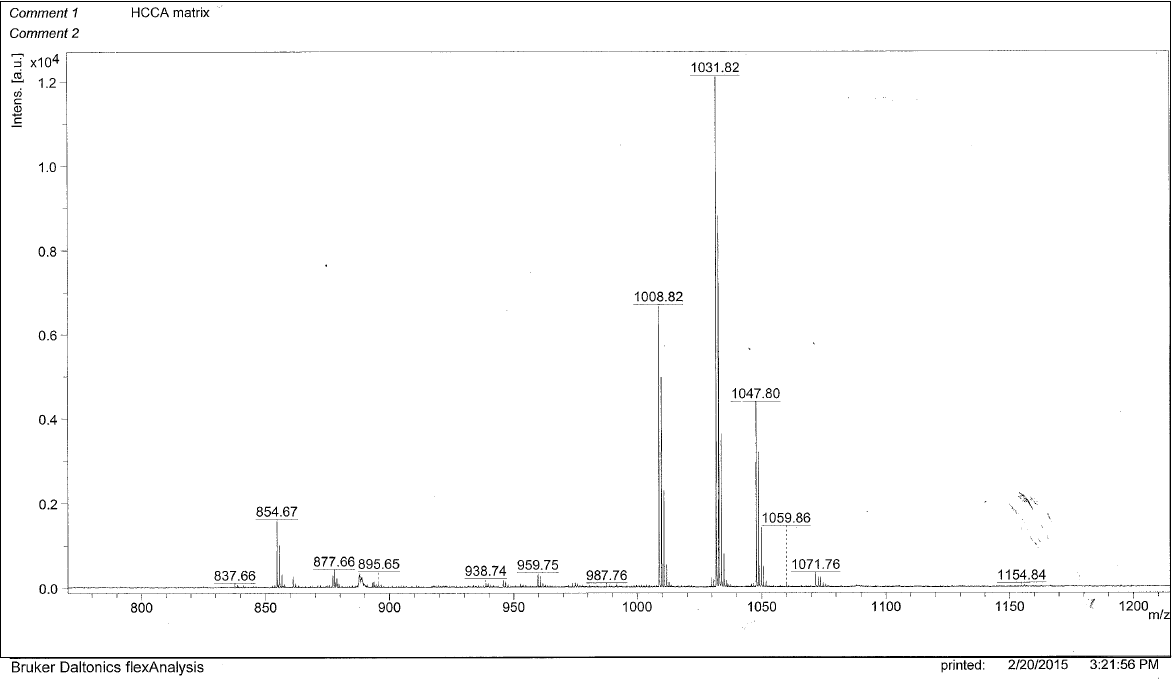
**

MALDI-TOF *N,N',N'',N'''*-(ethene-1,1,2,2-tetrayltetrakis(benzene-4,1-diyl))tetrakis(decanamide) **4**

**
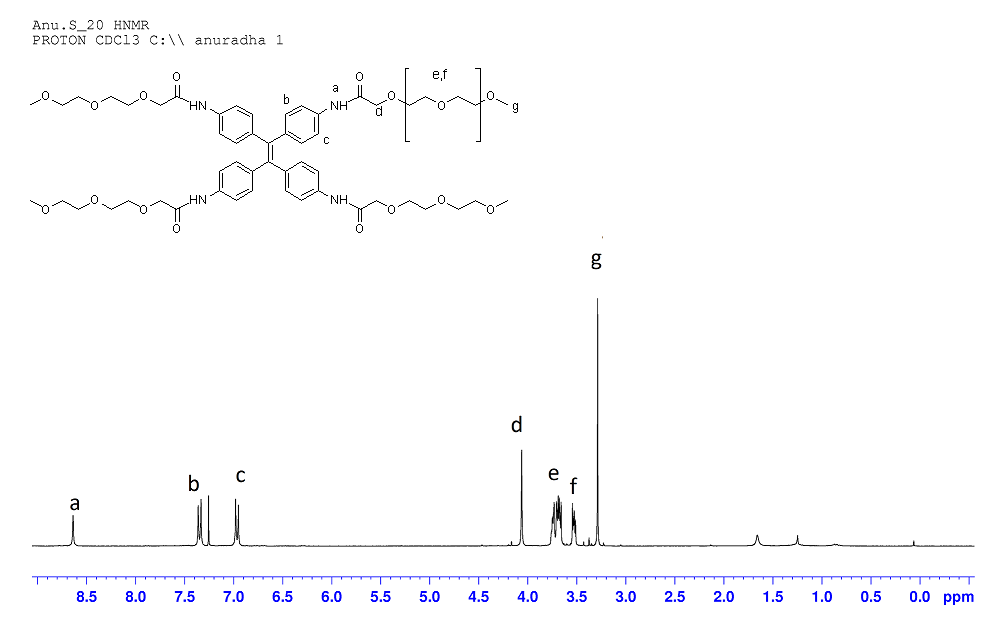
**

^1^HNMR *N,N',N'',N'''*-(ethene-1,1,2,2-tetrayltetrakis(benzene-4,1-diyl))tetrakis(2-(2-(2-methoxyethoxy)ethoxy)acetamide **6**

**
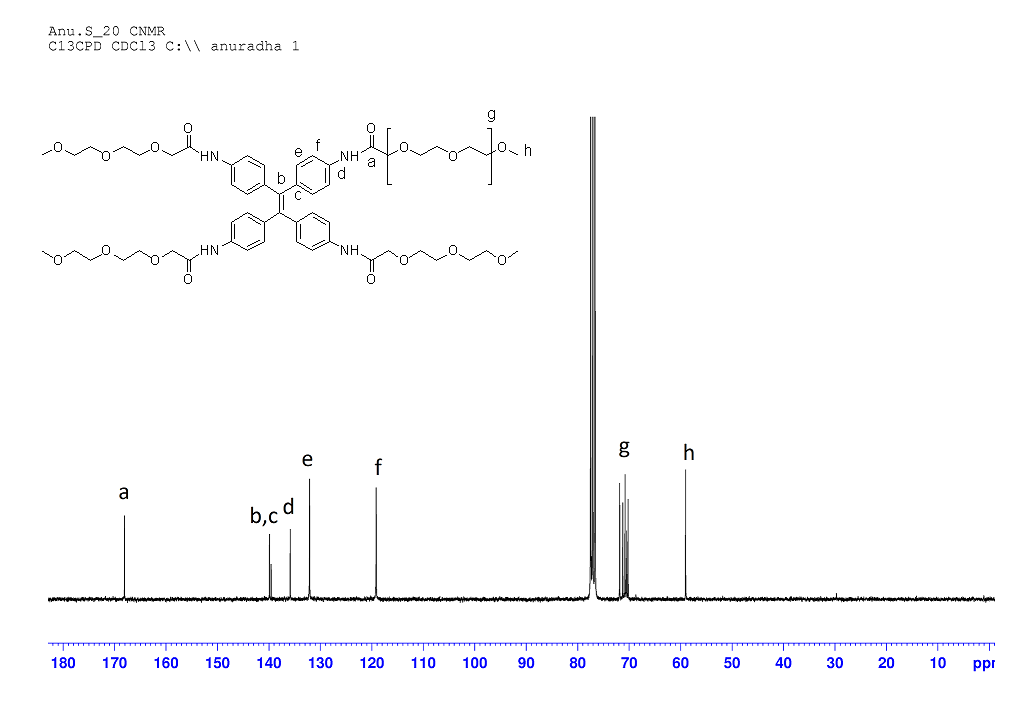
**

^13^CNMR *N,N',N'',N'''*-(ethene-1,1,2,2-tetrayltetrakis(benzene-4,1-diyl))tetrakis(2-(2-(2-methoxyethoxy)ethoxy)acetamide **6**

**
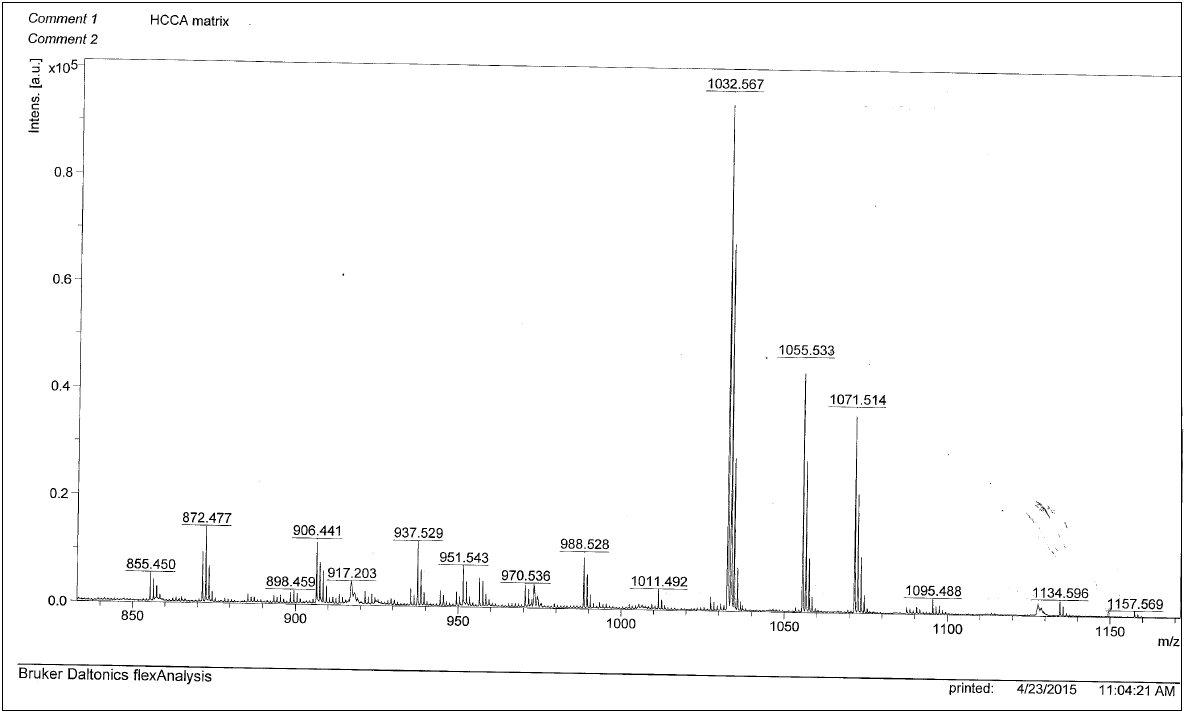
**

MALDI-TOF *N,N',N'',N'''*-(ethene-1,1,2,2-tetrayltetrakis(benzene-4,1-diyl))tetrakis(2-(2-(2-methoxyethoxy)ethoxy)acetamide **6**.
